# Photo-Cross-Linked Fullerene-Based Hole Transport Material for Moisture-Resistant Regular Fullerene Sandwich Perovskite Solar Cells

**DOI:** 10.1021/acsami.4c02573

**Published:** 2024-04-15

**Authors:** Andrea Cabrera-Espinoza, Silvia Collavini, José G. Sánchez, Ivet Kosta, Emilio Palomares, Juan Luis Delgado

**Affiliations:** † POLYMAT, University of the Basque Country UPV/EHU, Avenida Tolosa 72, Donostia/San Sebastián 20018, Spain; ‡ Institute of Chemical Research of Catalonia, The Barcelona Institute of Science and Technology (ICIQ-BIST), Avinguda Països Catalans 16, Tarragona 43007, Spain; § CIDETEC, Basque Research and Technology Alliance (BRTA), Paseo Miramón 196, Donostia/San Sebastián 20014, Spain; ∥ ICREA, Passeig Lluís Companys 23, Barcelona 08010, Spain; ⊥ Ikerbasque, Basque Foundation for Science, Bilbao 48013, Spain

**Keywords:** perovskite solar cells (PSCs), hole transport materials (HTMs), photo-cross-linking, moisture resistance, fullerene sandwich architecture

## Abstract

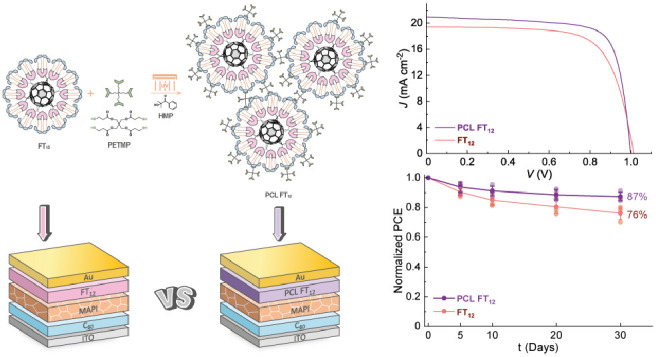

Despite the high efficiencies currently achieved with perovskite solar cells (PSCs), the need to develop stable devices, particularly in humid conditions, still remains. This study presents the synthesis of a novel photo-cross-linkable fullerene-based hole transport material named FT_12_. For the first time, the photo-cross-linking process is applied to PSCs, resulting in the preparation of photo-cross-linked FT_12_ (PCL FT_12_). Regular PSCs based on C_60_-sandwich architectures were fabricated using FT_12_ and PCL FT_12_ as dopant-free hole transport layers (HTLs) and compared to the reference spiro-OMeTAD. The photovoltaic results demonstrate that both FT_12_ and PCL FT_12_ significantly outperform pristine spiro-OMeTAD regarding device performance and stability. The comparison between devices based on FT_12_ and PCL FT_12_ demonstrates that the photo-cross-linking process enhances device efficiency. This improvement is primarily attributed to enhanced charge extraction, partial oxidation of the HTL, increased hole mobility, and improved layer morphology. PCL FT_12_-based devices exhibit improved stability compared to FT_12_ devices, primarily due to the superior moisture resistance achieved through photo-cross-linking.

## 1 Introduction

Perovskite solar cells (PSCs) represent a breakthrough in photovoltaics and are emerging as one of the most promising next-generation solar cell technologies. These remarkable devices employ ABX_3_ perovskite crystal structures as absorber materials, with “A” and “B” representing monovalent and divalent cations and “X” denoting a monovalent anion. Perovskites exhibit exceptional light-absorption capabilities, low exciton binding energies, high dielectric constants, outstanding electrical mobilities, and prolonged charge carrier lifetimes.^1^ These unique properties have enabled a maximum power conversion efficiency (PCE) of 26.1% in only 14 years of active research.^2^

Despite their impressive efficiency, PSCs face a critical challenge: the moisture stability of perovskite materials.^3^ The susceptibility of these devices to moisture can significantly impact their long-term performance and durability. Therefore, addressing this issue is crucial for advancing perovskite-based solar technology. Strategies, such as varying the ABX_3_ composition,^4^ introducing additives,^5 −12^ and advancing encapsulation techniques,^13^ have led to improvements in the stability of PSCs. Additionally, incorporating functional charge transport layers (CTLs), which shield the perovskite layer and exhibit exceptional electronic properties, has emerged as a promising approach to enhancing stability and boosting device efficiency.

In regular PSCs, light travels through a structure consisting of a transparent cathode, an electron transport layer (ETL), the perovskite layer, the hole transport layer (HTL), and, finally, a metal anode. The commonly used inorganic ETLs, titanium oxide (TiO_2_) and tin oxide (SnO_2_), have demonstrated suitable energy alignment with the perovskite conduction band, favorable electron mobility, and efficient optical transmittance. Nevertheless, both materials contain bulk defects that trap carriers, leading to charge recombination and reduced device efficiency and stability.^14,15^ Furthermore, the TiO_2_ layer exhibits high instability under UV irradiation and requires high-temperature sintering for preparation and the addition of dopants to enhance electron mobility.^14^ Fullerenes, such as C_60_ and its derivatives, are considered a promising alternative to inorganic electron transport materials (ETMs) due to their heightened UV resistance, demonstrated ability to passivate traps, and the lower processing temperature required for thin-film deposition from solution.^16−22^

Regarding HTLs, spiro-OMeTAD (2,2′,7,7′-tetrakis[*N*,*N*-di(4-methoxyphenyl)amino]-9,9′-spirobifluorene) has been the most commonly employed standard and remains the primary hole transport material (HTM) employed in regular PSCs.^23^ The high device efficiencies achieved with spiro-OMeTAD result from the enhanced alignment of its highest occupied molecular orbital (HOMO) with the perovskite valence band and the improved hole mobility, attributes that can only be achieved through doping, given that pristine spiro-OMeTAD exhibits significant resistance to hole conduction. Typically, spiro-OMeTAD is doped with bis(trifluoromethane)sulfonimide lithium salt (Li-TFSI), tris(2-(1H-pyrazol-1-yl)-4-*tert*-butylpyridine)cobalt(II) di[bis(trifluoromethane) sulfonimide] (FK209), and 4-*tert*-butylpyridine (*t*-BP).^23^ However, the incorporation of these dopants has been associated with reduced long-term stability in PSCs due to two primary factors: their migration within the perovskite layer and their hygroscopic nature. The latter is particularly noticeable in the case of Li-TFSI, which accelerates moisture-induced degradation of the perovskite layer.^24^

To mitigate potential damage to the perovskite layer and eliminate dependence on dopants, the development of alternative HTMs with efficient electronic properties that do not require these additives is highly desirable for PSC applications.^25,26^

In this context, our research group introduced FU7 as the first fullerene-based HTM that exhibited substantial hole mobility and suitable energy alignment with the perovskite layer without doping.^27^ The chemical structure of FU7 consisted of a hexakis-C_60_ core and 12 methoxy-terminated π-extended triarylamine units. The hexafunctionalization was employed to achieve a lower unoccupied molecular orbital (LUMO) energy level capable of blocking electrons from the perovskite, while the incorporation of π-extended triarylamine units was motivated by reported improvements in hole mobility and the achievement of higher PCE in PSCs when compared to the use of simple triphenylamine-based HTMs.^28^

Photovoltaic results demonstrated that incorporating undoped FU7 into regular PSCs resulted in a significantly higher PCE than the efficiency achieved with undoped spiro-OMeTAD. Furthermore, this work introduced the innovative design of a C_60_-sandwich PSC structure, representing a novel architecture where both the ETL and HTL are based on C_60_-fullerene materials (C_60_ and FU7). In this configuration, an initial PCE of about 9% was achieved.^27^

**Figure 1:**
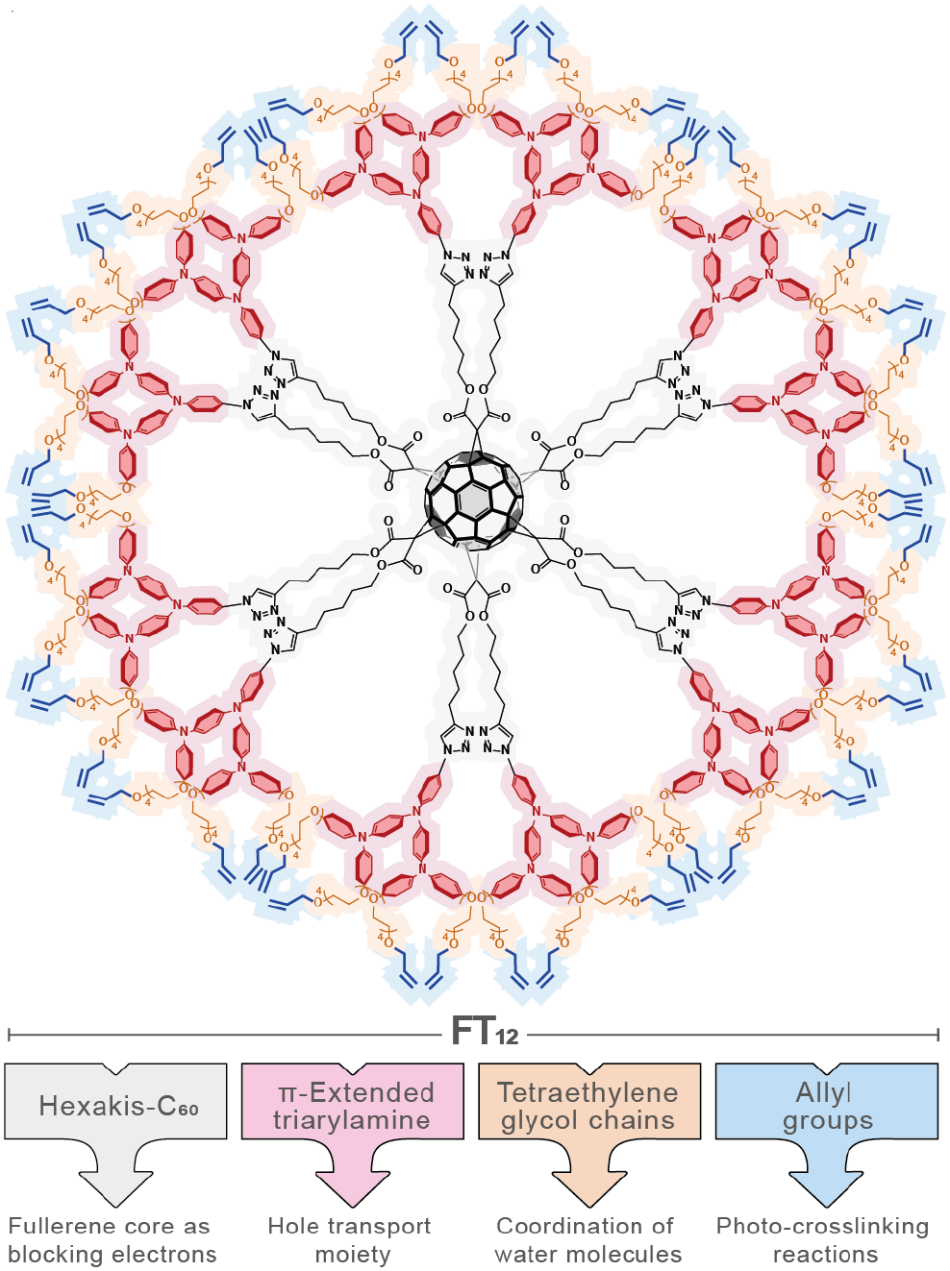
Chemical structure of FT_12_.

Further advancements in the utilization of C_60_ derivatives as HTLs have been achieved in this study through the synthesis and application of FT_12_. Similar to FU7, the chemical composition of FT_12_ is derived from hexakis-C_60_ functionalized with 12 π-extended triarylamine units. However, two additional functional groups, comprising tetraethylene glycol and allyl moieties, have been incorporated, as depicted in Figure 1.

Tetraethylene glycol chains have been incorporated into the chemical structure of FT_12_ to increase device stability. These chains have been shown to coordinate with water molecules through hydrogen bonding, attaching them to the surface of the ethylene glycol material and inhibiting water infiltration into the perovskite layer.^6,29^

On the other hand, the introduction of allylic groups in FT_12_ was proposed to induce photo-cross-linking reactions, leading to the formation of a new material referred to as photo-cross-linked FT_12_ (PCL FT_12_). To the best of our knowledge, the photo-cross-linking approach has not yet been used in the design of top HTLs for PSCs, possibly due to the adverse effects of UV irradiation.^30^ Nevertheless, some existing literature has also reported the advantages of utilizing short UV exposure times at high power intensity to improve solar cell efficiency and enhance hole conductivity in triarylamine-based HTLs.^31−34^

The higher reactivity of allyl groups compared to other unsaturated functional groups employed in thiol–ene photo-cross-linking has been previously reported.^35^ Likewise, the advantages of introducing thiol pentaerythritol tetrakis(3-mercaptopropionate) (PETMP) into PSCs have been demonstrated.^36−38^

In this context, along with the investigation related to the utilization of FT_12_, this study includes a comparative analysis between the use of non-cross-linked FT_12_ and photo-cross-linked PCL FT_12_ obtained through a reaction with the thiol PETMP. This comparison aims to evaluate the effects of the photo-cross-linking process on the efficiency and stability of regular PSCs.

Considering the enhanced stability of C_60_ under UV irradiation in contrast to conventional inorganic ETLs, both FT_12_ and PCL FT_12_ were integrated into C_60_-sandwich PSC architectures. These devices followed the ITO/C_60_/MAPI/HTL/Au structure, where MAPI refers to methylammonium lead iodide perovskite (MAPbI_3_).

## 2 Results and Discussion

### 2.1 Synthesis and Characterization of FT_12_

**Scheme 1:**
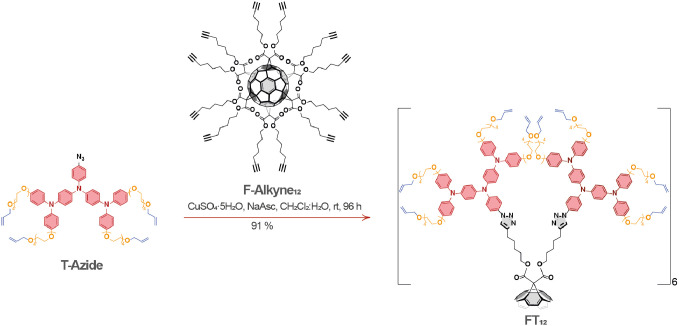
Synthesis of FT_12_

FT_12_ was synthesized by a click reaction with T-Azide and F-Alkyne_12_, as illustrated in Scheme 1. The preparation of T-Azide and F-Alkyne_12_ was performed according to Schemes S1 and S2, as detailed in the Supporting Information. The click reaction is based on the Huisgen 1,3-dipolar cycloaddition and was carried out in a catalytic medium consisting of CuSO_4_·5H_2_O/sodium ascorbate (NaAsc), according to a previously reported protocol.^39^ Comprehensive synthetic details can be found in the Experimental Section.

The chemical structure of FT_12_ was confirmed by NMR and FTIR spectroscopy. The ^1^H NMR spectrum in Figure S19 highlights the singlet typical of the triazole proton at 7.3 ppm and the signals corresponding to the allylic protons at 5.3, 5.1, and 5.9 ppm. The ^13^C NMR spectrum in Figure S20 displays all the expected signals for the proposed chemical structure. Among the most representative signals are those involving the carbon signal for the carbonyl group at 163.7 ppm, the signal at 45.3 ppm assigned to the bridging carbon, and the signal at 72.4 ppm corresponding to the *sp*^3^-hybridized carbon atom in the fullerene cage.^39^

When comparing the FTIR spectrum of FT_12_ with those of its precursors, as shown in Figure S21a, it is evident that the characteristic N≡N stretching absorption of the azide group at 2088 cm^–1^ in T-Azide has wholly disappeared,^40^ as have the absorption bands at 3289 and 2115 cm^–1^, which are characteristic of the terminal alkyne group in F-Alkyne.^40^ These observations confirm the successful completion of the click 1,3-dipolar cycloaddition between F-Alkyne and T-Azide. Furthermore, a MALDI-TOF mass spectrum was obtained (Figure S21b) to validate the acquisition of FT_12_, highlighting the correspondence between the *m*/*z* value of the molecular ion peak and its molecular weight.

**Figure 2:**
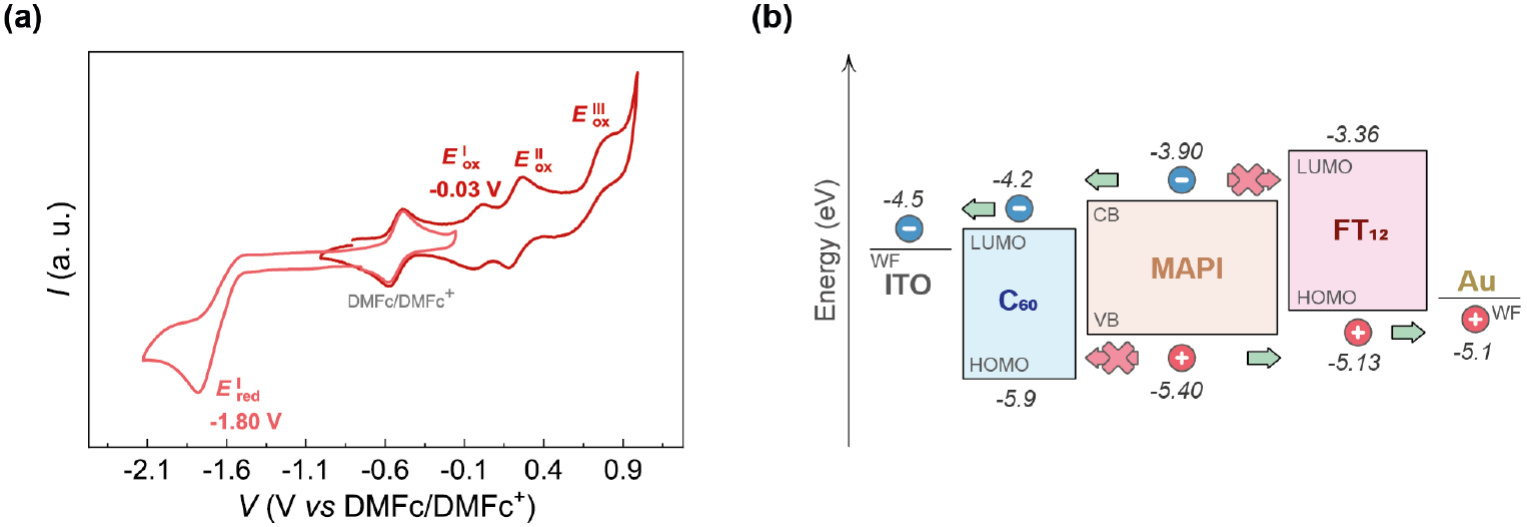
(a) Cyclic voltammogram of FT_12_ and half-wave potential for the first reduction () and first oxidation (). (b) Energy diagram of a PSC using FT_12_ as HTL. The energy levels of the remaining components were assigned as reported in the literature.^43,44^

Cyclic voltammetry was employed for the evaluation of electrochemical behavior and estimation of the HOMO and LUMO energy levels. Comprehensive cell preparation and calculation information can be found in the Supporting Information. Figure 2a reveals one irreversible reduction process and three distinct quasi-reversible oxidations observed within the specified electrochemical window. These results are consistent with previous reports describing the reduction process of the C_60_ hexakis-adduct and the oxidation processes of π-extended triarylamines with three redox nitrogen centers.^41^

The LUMO and HOMO energy levels were estimated to be −3.36 and −5.13 eV, respectively, using the first reduction process () and the first oxidation process (), as determined by the equation *E*_LUMO/HOMO_ = .^42^Figure 2b illustrates the energy levels of a PSC with an architecture based on ITO/C_60_/MAPI/FT_12_/Au.^43,44^ The high LUMO energy level of FT_12_ is observed to hinder electron transfer from the perovskite, while the positioning of the HOMO energy level in FT_12_ facilitates hole transfer to the anode. These observations confirm that FT_12_ exhibits the requisite energetic characteristics to function as a suitable HTM for PSCs.

### 2.2 Photo-Cross-Linking Process

**Figure 3:**
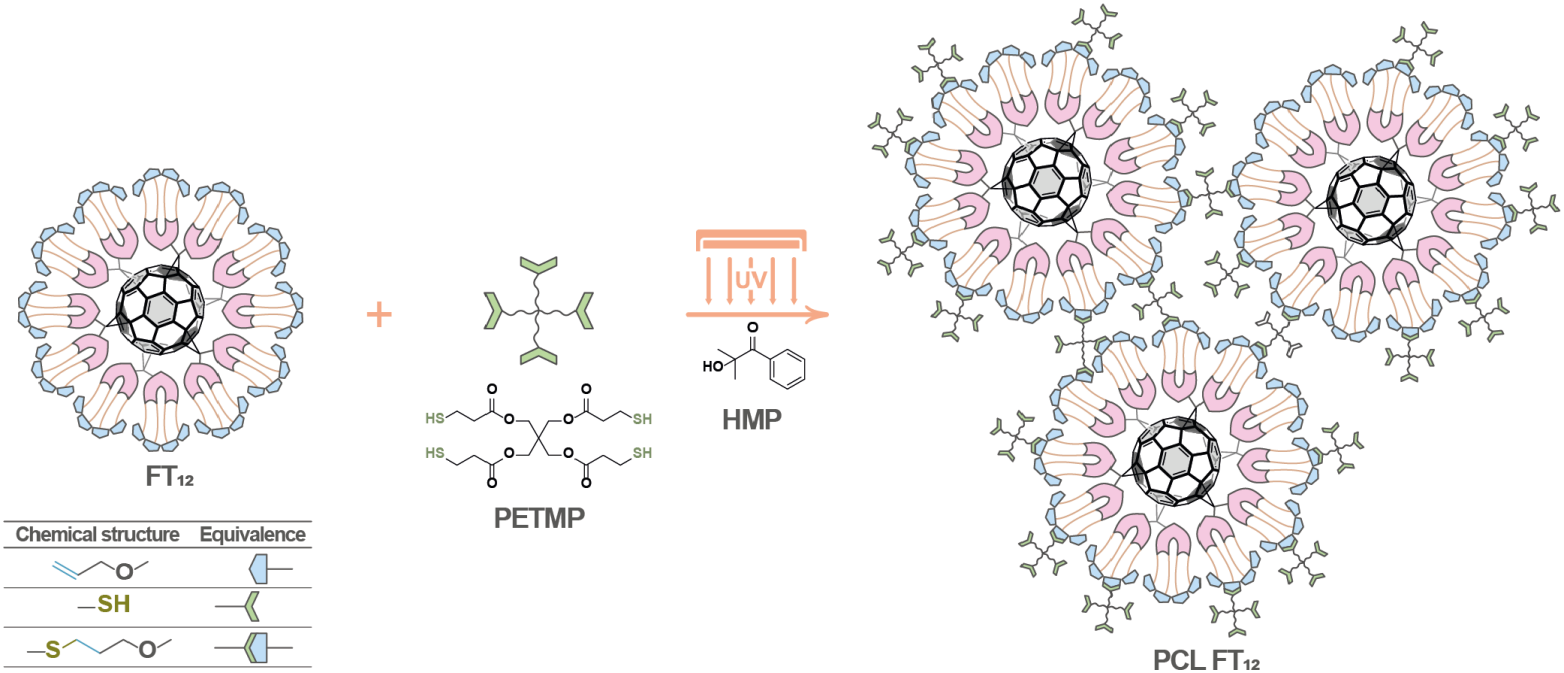
Photo-cross-linking reaction.

Following the synthesis and characterization of FT_12_, the photo-cross-linking reaction was carried out using the thiol pentaerythritol-tetrakis(3-mercaptopropionate) (PETMP) and the photoinitiator 2-hydroxy-2-methylpropiophenone (HMP) (Figure 3). PETMP was selected for its high selectivity with the allyl groups and the demonstrated benefits of using this thiol in PSCs.^36−38^ On the other hand, HMP was chosen as the photoinitiator because of its ability to generate radicals rapidly and its high solubility in orthogonal solvents for perovskite, which facilitates its removal after the photo-cross-linking process.^45^

**Figure 4:**
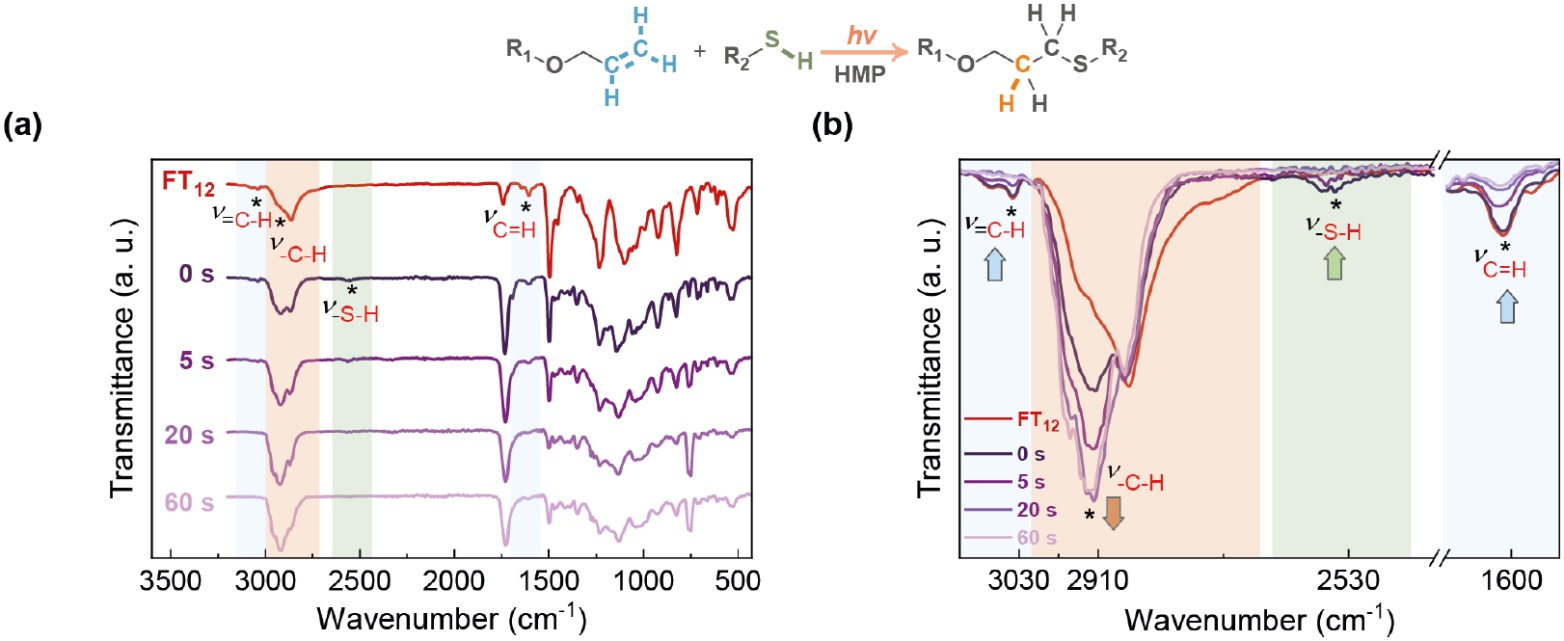
FTIR spectra for the photo-cross-linking reaction (inset) under different UV exposure times: (a) complete and (b) enlarged spectra.

Under these conditions, the initial steps involve the formation of radicals from the photoinitiator upon UV irradiation. These radicals then abstract a hydrogen atom from the thiol, resulting in the formation of thiyl radicals. Subsequently, the thiyl radicals undergo addition reactions to the C=C double bonds of the allyl group, forming alkyl radicals. Next, new C–H bonds are formed as the alkyl radical abstracts a hydrogen atom from the unreacted thiol groups, as proposed by reaction mechanisms reported in the literature.^46^ The formation of PCL FT_12_ is depicted in the reaction scheme in Figure 4.

The photo-cross-linking reaction was evaluated by FTIR spectroscopy. Details of the photo-cross-linking procedure are given in the Experimental Section. To prepare the samples, a solution containing FT_12_, PETMP, and HMP was drop-cast onto an aluminum disk. After drying, the resulting films were exposed to UV light for 0, 5, 20, and 60 s.

As depicted in Figure 4, a comparison between the FT_12_ spectrum (in red) and the spectra from the mixture (in purple) reveals changes induced by UV irradiation. Special attention was directed toward regions around 3030 and 1600 cm^–1^ (in blue), corresponding to =C–H and C=H stretching vibrations of the allyl groups, the region around 2530 cm^–1^ associated with the S–H stretching mode of the thiol (in green), and the region around 2910 cm^–1^ assigned to alkane C–H stretching vibrations (in orange).^47,48^

As shown in the spectra, the absorption intensities of the allyl and thiol groups progressively decreased, while the absorption intensity of C–H increased with UV irradiation. Considering the bonds involved in the cross-linking reaction mentioned above, this study confirms the successful photo-cross-linking between FT_12_ and PETMP. Given the minimal changes observed between 20 and 60 s, it can be deduced that 20 s of UV irradiation is sufficient to complete the cross-linking reaction.

**Figure 5:**
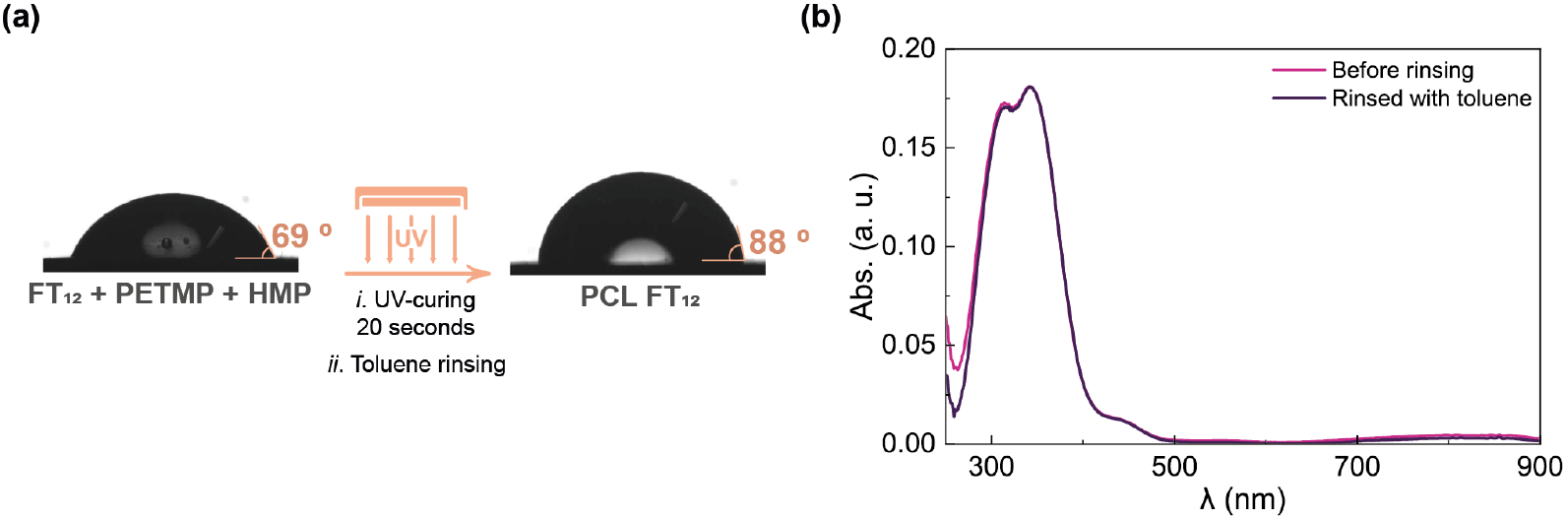
(a) Water contact angles on the surfaces of the photo-cross-linking reaction mixture before and after UV-curing and rinsing. (b) UV–vis spectra of the PCL FT_12_ film before and after being rinsed with toluene.

Contact angle measurements evaluated the surface wettability of PCL FT_12_. The sample preparation procedure involved spin-coating the reaction mixture onto glass substrates, followed by UV-curing for 20 s. Subsequently, toluene was dynamically spin-coated onto the surface to remove any residual reagents in accordance with suggestions from the literature.^38^Figure 5a presents the contact angles of the mixture before and after UV exposure, revealing an increase from 69° to 88°. This significant change indicates that the photo-cross-linked film (PCL FT_12_) exhibits greater hydrophobicity than the non-cross-linked (FT_12_). Statistical contact angle data can be found in Figure S22.

The resistance to toluene rinsing was evaluated by measuring the UV–vis absorbance of the PCL FT_12_ film before and after rinsing. Figure 5b demonstrates that the spectra exhibit minor variations, indicating the high resistance of PCL FT_12_ to this solvent.

Furthermore, Figure S23 presents images extracted from videos depicting the dynamic movement of a water droplet on both PCL FT_12_ and the surface obtained using the photo-cross-linking reaction mixture (FT_12_ + PETMP + HMP) (Videos S1 and S2). These images demonstrate how the cross-linked surface of PCL FT_12_ repels water, inhibiting its dispersion across the film and conserving a liquid-free surface, in contrast to the behavior observed with the non-cross-linked film.

### 2.3 Perovskite Solar Cells with FT_12_ and PCL FT_12_

**Figure 6:**
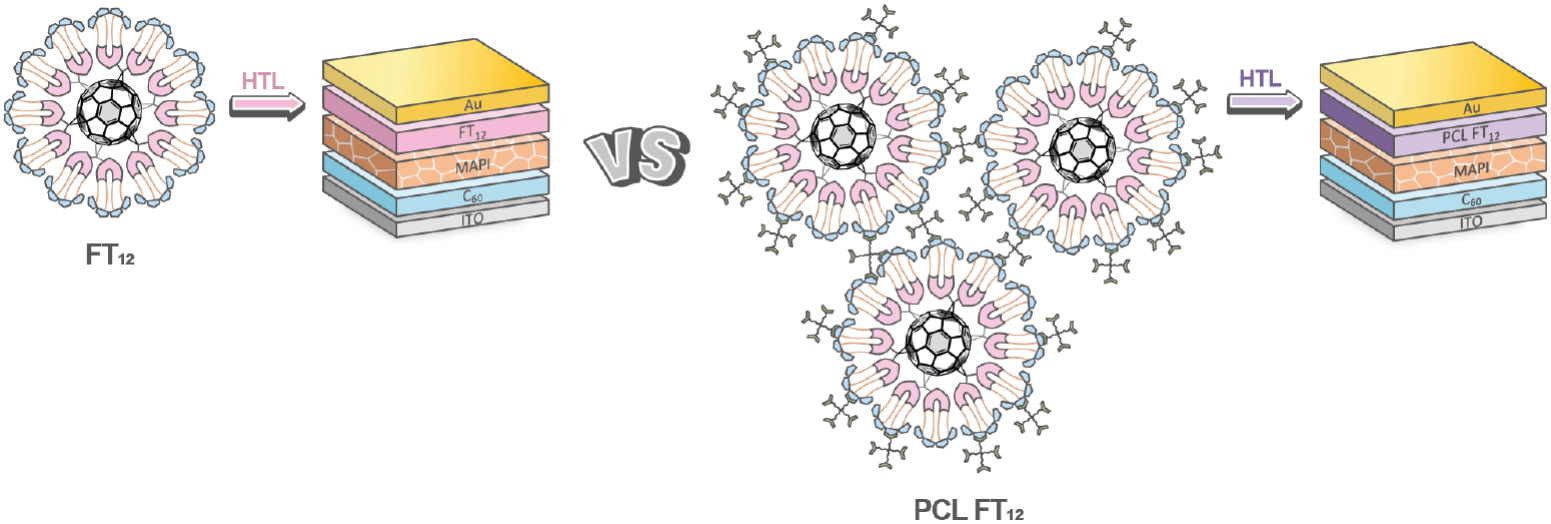
C_60_-sandwich PSCs based on FT_12_ and PCL FT_12_ as HTLs.

A comparison was performed to evaluate the impact of the photo-cross-linking process on PSCs with a non-cross-linked FT_12_ and those with a cross-linked PCL FT_12_. These devices were fabricated using a C_60_-sandwich PSC architecture, with FT_12_ and PCL FT_12_ serving as dopant-free HTLs, as illustrated in Figure 6.

#### 2.3.1 Fabrication and Characterization of PSCs

All the layers were prepared using the spin-coating technique on prepatterned ITO glass substrates, whereas the gold contact was deposited through thermal evaporation. The C_60_ and MAPI layers were prepared according to the optimizations and recommendations reported in the literature.^43,49 −53^ Detailed information regarding device fabrication can be found in the Experimental Section.

Before comparing FT_12_ and PCL FT_12_-based PSCs, the optimal concentration of FT_12_ was determined by fabricating FT_12_-based PSCs using precursor solutions with concentrations ranging from 8 to 15 mg/mL. Analysis of the current density–voltage (*J–V*) curves and statistical data of cell parameters (Figure S24 and Table S1) indicated that the appropriate concentration was 11 mg/mL.

To implement the photo-cross-linking process in the design of PSCs, an initial optimization of the amount of PETMP was performed. Precursor solutions consisting of a mixture of 11 mg/mL FT_12_ and various concentrations of PETMP ranging from 0.5 to 5.0 mol equiv was prepared for the fabrication of HTLs. The *J–V* curves of the highest-performing PSCs and the statistical data of the photovoltaic parameters (Figure S25 and Table S2) reveal that the short-circuit current density (*J*_SC_) and the fill factor (FF) increased slightly upon the incorporation of 1 equiv of PETMP. However, both decreased at higher concentrations of PETMP. The open circuit voltage (*V*_OC_) decreased somewhat between 1 and 3.0 equiv of PETMP, with a more significant drop observed at 5.0 equiv, a concentration that also affected the other photovoltaic parameters.

The improvements in device performance upon the inclusion of 0.5 and 1.0 equiv of PETMP may be attributed to enhanced interfaces with the perovskite layer, a phenomenon previously demonstrated when employing this thiol derivative as an interlayer between perovskite/charge transport layer (CTL) and additive in the CTLs.^36−38^ Based on these results, a concentration of 1 equiv of PETMP to the FT_12_ concentration was selected, ensuring this value was not exceeded.

A precursor solution comprising a mixture of 11 mg/mL FT_12_, 1.0 equiv of PETMP, and 1% w/w HMP was deposited onto the perovskite layer to obtain the photo-cross-linked PSCs. The amount of HMP used followed previously reported recommendations.^54^ After drying, the film was UV-cured for 20 s, rinsed in toluene, and gold was evaporated to complete the device.

**Figure 7:**
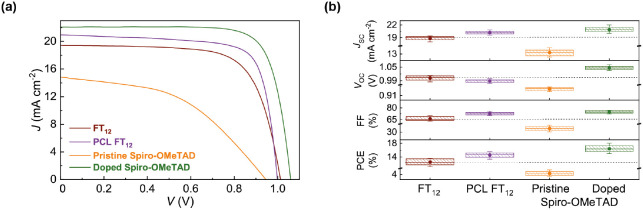
(a) *J*–*V* curves of the best-performing PSCs using FT_12_, PCL FT_12_, pristine, and doped spiro-OMeTAD as HTLs. (b) Statistical box plots of the photovoltaic parameters from around 15 devices. The dashed line on the graph has been positioned at the average values obtained using FT_12_.

To verify the appropriate preparation of PSCs, control devices were fabricated using doped spiro-OMeTAD. In addition, to evaluate the effect of doping on the performance of devices containing spiro-OMeTAD, PSCs based on pristine spiro-OMeTAD were also fabricated. Figure 7 displays the *J*–*V* curves for the best-performing devices using the different HTLs (FT_12_, PCL FT_12_, pristine, and doped spiro-OMeTAD), accompanied by the statistical analysis of cell parameters derived from approximately 15 devices using each HTL. All parameters are summarized in Table 1.

**Table 1:** Best and Average Photovoltaic Parameters of PSCs Using FT_12_, PCL FT_12_, Pristine, and Doped Spiro-OMeTAD as HTLs^a^^b^

HTL	*J*_SC_(mA cm^–2^)	*V*_OC_(V)	FF (%)	PCE (%)	*R*_s_(Ω cm^2^)	*R*_sh_(kΩ cm^2^)
FT_12_	19.4 (18.9 ± 0.5)	1.01 (1.00 ± 0.01)	70 (66 ± 3)	13.7 (12.5 ± 0.9)	6.8 (7.3 ± 0.5)	1.74 (1.68 ± 0.05)
PCL FT_12_	20.8 (20.2 ± 0.4)	1.00 (0.98 ± 0.02)	75 (73 ± 2)	15.5 (14.6 ± 0.7)	3.8 (4.4 ± 0.6)	0.87 (0.79 ± 0.09)
Pristine Spiro-OMeTAD	14.5 (13.4 ± 0.8)	0.95 (0.94 ± 0.01)	39 (35 ± 3)	5.3 (4.4 ± 0.6)	28.2 (30.1 ± 0.6)	0.22 (0.15 ± 0.08)
Doped Spiro-OMeTAD	22.0 (20.9 ± 0.6)	1.06 (1.05 ± 0.02)	77 (75 ± 2)	18.0 (16.0 ± 1.0)	4.8 (6.1 ± 0.9)	3.21 (3.15 ± 0.06)

a Statistical results obtained from around 15 devices per condition.

b The values of the series resistances (*R*_s_) were determined from the inverse slope of the *J*–*V* curve near the open-circuit voltage. In contrast, the shunt resistances (*R*_sh_) were determined from the inverse slope near the short-circuit current density.

The control device using doped spiro-OMeTAD exhibited a maximum power conversion efficiency (PCE) of 18.0%, with a *V*_OC_ of 1.06 V, a *J*_SC_ of 22.0 mA cm^–2^, and a FF of 77%, similar to the reported efficiencies in the literature for C_60_/MAPI/spiro-OMeTAD-based architectures.^43,44^ The impact of doping was evident when compared to PSCs based on pristine spiro-OMeTAD, which demonstrated the lowest PCEs of 5.3% due to reduced *J*_SC_, *V*_OC_, and FF. These results are consistent with literature findings that attribute the reduced efficiency to the limited hole mobility and conductivity of pristine spiro-OMeTAD, resulting in enhanced hole accumulation and carrier recombination within the device.^55^

When comparing the use of pristine HTLs, the incorporation of FT_12_ and PCL FT_12_ leads to substantial enhancements in all photovoltaic parameters compared to pristine spiro-OMeTAD, highlighting the potential of these innovative organic materials for PSC applications. A comparison between PSCs utilizing FT_12_ and those PCL FT_12_ revealed that the photo-cross-linking process improves the *J*_SC_ and FF, enhancing device performance from 13.7% to 15.5%. It is worth noting that both of these efficiencies exceed the previous reports in the literature with FU7.

External quantum efficiency (EQE) measurements further validated the *J*_SC_ values. Figure S26 demonstrates that the integrated *J*_SC_ values for the PCL FT_12_ and FT_12_ devices are 19.8 and 18.5 mA cm^–2^, respectively, consistent with the values extracted from the *J–V* curves with a deviation of approximately 6%.

Analyzing the series resistances (*R*_s_) shown in Table 1, it is observed that the photo-cross-linking process reduced *R*_s_ from 6.8 to 3.8 Ω cm^–2^. Remarkably, devices utilizing PCL FT_12_ exhibited lower *R*_s_ values than those employing the optimized doped spiro-OMeTAD (4.8 Ω cm^–2^). The decrease in *R*_s_ has been attributed to improved interfacial contact between the perovskite layer and the HTL. This leads to enhanced hole extraction by the HTL, which contributes to an increase in FF by reducing interfacial charge recombination.^56,57^

**Figure 8:**
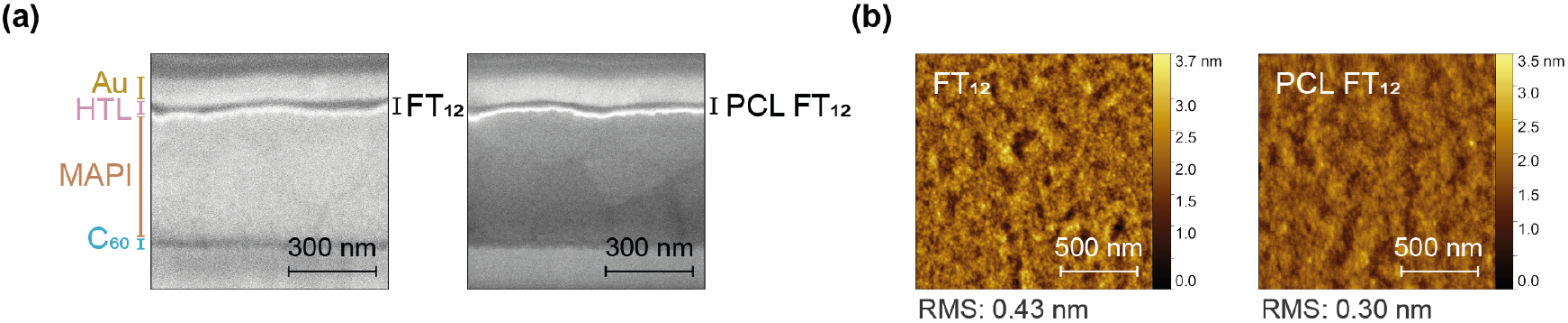
(a) Cross-sectional FESEM images of PSCs based on FT_12_ and PCL FT_12_ and (b) AFM topographic images of FT_12_ and PCL FT_12_ layers.

Field emission scanning electron microscopy (FESEM) measurements and atomic force microscopy (AFM) topography analysis were performed to evaluate the effect of HTL morphology on device performance. Figure 8a shows that both FT_12_ and PCL FT_12_ have comparable thicknesses of 52 and 45 nm, respectively, providing complete coverage of the perovskite layer. Notably, in Figure 8b, the AFM topographic images reveal that in both cases, the films are homogeneous and that the photo-cross-linking process reduces the root-mean-square (RMS) roughness value from 0.43 to 0.30 nm, indicating a smoother film morphology in PCL FT_12_ compared to FT_12_. This enhancement in HTL quality is likely associated with improved interfacial contact and, consequently, influences the improvements in FF in PCL FT_12_-based devices.^57^

Photoluminescence (PL) measurements on MAPI/HTL substrates were conducted to analyze the variations in interfacial charge extraction when using FT_12_ and PCL FT_12_. Figure S26 presents the steady-state PL spectra and the time-resolved PL (TRPL) lifetime curves. Information regarding substrate preparation and the calculation of the average carrier lifetimes (τ_avg_) are provided in the Supporting Information.

Figure S27a shows a significant reduction in the PL emission intensity of the MAPI film when these HTLs are applied to its surface, indicating effective hole extraction at the MAPI/HTL interfaces. The more significant reduction in intensity observed with PCL FT_12_ incorporation suggests more efficient charge extraction and transport than with FT_12_. This observation is further supported by the TRPL curves in Figure S27b, where fitting analysis reveals shorter τ_avg_ values after the photo-cross-linking process (56.9 s for PCL FT_12_ vs 75.7 s for FT_12_). These results indicate a significant improvement of electronic contact interface properties in PCL FT_12_ compared to FT_12_. This improvement leads to a faster charge extraction at the perovskite interface with PCL FT_12_, which could explain the increased *J*_SC_ and FF observed in the *J–V* curves.^58,59^

In addition, PCL FT_12_ exhibits a reduction in *R*_sh_ and *V*_OC_ compared to FT_12_ (Figure 7 and Table 1). Previous studies on the impact of UV irradiation on PSCs have demonstrated that UV exposure reduces *R*_sh_ primarily due to the partial degradation of the perovskite layer, while *V*_OC_ is affected by changes in the energy levels of PSC components caused by UV treatment.^31,32,60^

**Figure 9:**
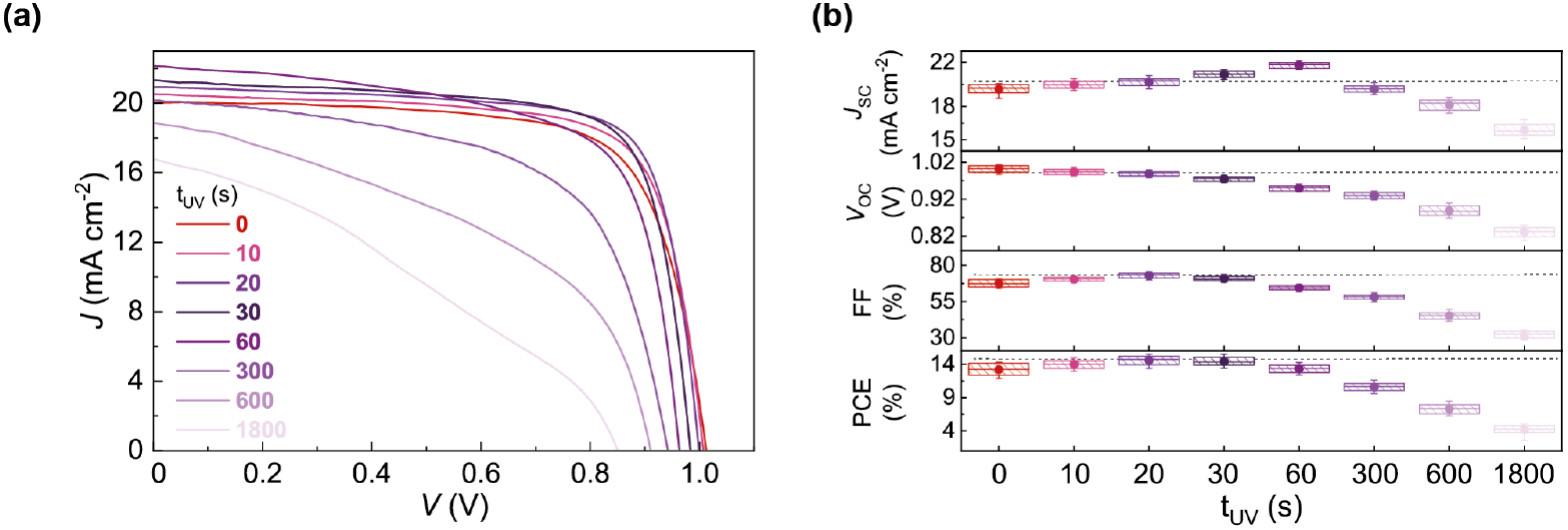
(a) *J*–*V* curves of the best-performing PSCs at different UV exposure times (*t*_UV_) for PCL FT_12_ film preparation. (b) Statistical box plots of the photovoltaic parameters from around 15 devices. The dashed line on the graph has been positioned at the average values obtained at 20 s.

Therefore, to evaluate the effect of UV irradiation on our photo-cross-linked device, the UV curing time during the preparation of PCL FT_12_ was varied from 0 to 1800 s. Figure 9 displays the *J–V* curves for the best-performing devices at various UV irradiation times and the statistical analysis of the principal photovoltaic parameters obtained from 10 to 15 devices per condition. All results are summarized in Table 2.

**Table 2:** Best and Average Photovoltaic Parameters of PSCs at Different UV Exposure Times (*t*_UV_) for PCL FT_12_ Film Preparation^a^^b^

*t*_UV_ (s)	*J*_SC_ (mA cm^–2^)	*V*_OC_ (V)	FF (%)	PCE (%)	*R*_s_ (Ω cm^2^)	*R*_sh_ (kΩ cm^2^)
0	20.1 (19.6 ± 0.5)	1.01 (1.00 ± 0.01)	71 (67 ± 3)	14.3 (13.2 ± 0.9)	5.5 (6.1 ± 0.4)	1.29 (1.20 ± 0.07)
10	20.5 (20.0 ± 0.4)	1.01 (0.99 ± 0.01)	72 (70 ± 2)	15.0 (14.0 ± 0.7)	4.4 (5.0 ± 0.7)	1.05 (0.92 ± 0.07)
20	20.8 (20.2 ± 0.4)	1.00 (0.98 ± 0.02)	75 (73 ± 2)	15.5 (14.6 ± 0.7)	3.8 (4.4 ± 0.6)	0.87 (0.79 ± 0.09)
30	21.3 (20.9 ± 0.3)	0.98 (0.98 ± 0.01)	74 (73 ± 2)	15.5 (14.4 ± 0.7)	3.7 (4.2 ± 0.4)	0.70 (0.60 ± 0.08)
60	22.2 (21.8 ± 0.3)	0.96 (0.95 ± 0.01)	67 (65 ± 2)	14.3 (13.3 ± 0.6)	4.1 (4.9 ± 0.8)	0.33 (0.27 ± 0.05)
300	20.2 (19.6 ± 0.4)	0.94 (0.93 ± 0.01)	61 (58 ± 2)	11.6 (10.6 ± 0.7)	7.8 (8.3 ± 0.4)	0.21 (0.16 ± 0.06)
600	18.8 (18.1 ± 0.5)	0.91 (0.89 ± 0.01)	50 (45 ± 3)	8.5 (7.3 ± 0.8)	11.0 (11.8 ± 0.6)	0.10 (0.07 ± 0.04)
1800	16.8 (15.9 ± 0.6)	0.85 (0.83 ± 0.01)	36 (31 ± 4)	5.1 (4.1 ± 0.8)	19.2 (20.1 ± 0.9)	0.07 (0.02 ± 0.05)

a Statistical results obtained from around 15 devices per condition.

b The values of the series resistances (*R*_s_) were determined from the inverse slope of the *J*–*V* curve near the open-circuit voltage. In contrast, the shunt resistances (*R*_sh_) were determined from the inverse slope near the short-circuit current density. The devices at 0 s correspond to the PSCs with HTLs based on the photocross-linking reaction mixture of FT_12_, PETMP, and HMP without UV curing and rinsing.

The results presented in Figure 9 and Table 2 reveal significant effects on each cell parameter with UV exposure time. Both *V*_OC_ and *R*_sh_ gradually decrease with increasing UV curing time, confirming the correlation between these parameters and the damaging effect of UV irradiation on the PSCs. The continuous decrease of these parameters is likely a consequence of the perovskite degradation and changes in the energy levels of the components of PSCs due to prolonged UV exposure, as reported in the literature.^31,32^

On the other hand, UV exposure is beneficial for the other parameters in the initial stages. During the first 20 s of exposure, *R*_s_ values decrease, leading to an increase in FF. This suggests a reduction in charge recombination and an enhancement in hole transfer at the perovskite/HTL interface due to the UV treatment.^31^ However, beyond this point, FF begins to decline due to increasing *R*_s_, indicating that prolonged UV exposure could affect the charge recombination and transport processes at the interfaces. Additionally, *J*_SC_ rises up to the 60-s threshold, after which it gradually declines. The reason for the increase in the *J*_SC_ will be explained in Section 2.3.2.

Despite the increase in *J*_SC_ up to 60 s, the adverse effects on the FF after 20 s and the decline in *V*_OC_ are accountable for the limited increase in PCE, observed only up to 20 s.

#### 2.3.2 Electronic Properties of FT_12_ and PCL FT_12_

**Figure 10:**
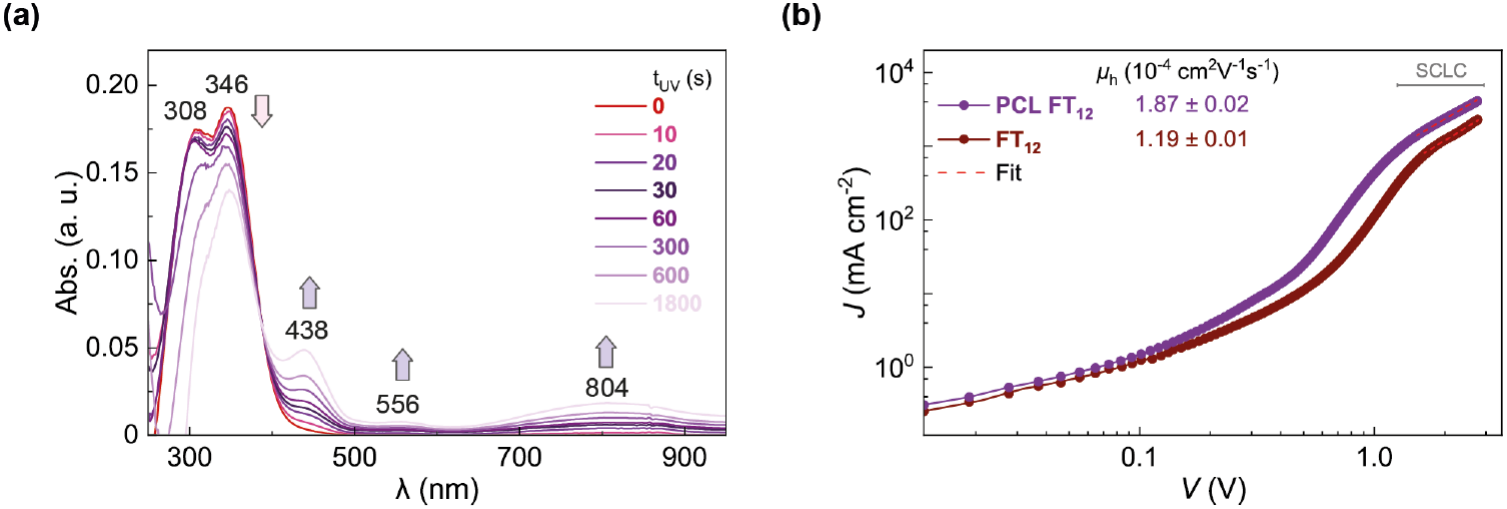
(a) UV–vis absorption spectra of films based on photo-cross-linking reaction mixtures at different UV exposure times (*t*_UV_) and (b) hole mobilities (μ_h_) of FT_12_ and PCL FT_12_ layers.

To evaluate the effect of UV irradiation on the electronic properties of PCL FT_12_, films based on the photo-cross-linking reaction mixture were exposed to UV radiation at various times (*t*_UV_), followed by the measurement of UV–vis absorption spectra. Figure 10a shows that upon longer UV exposure, the absorption intensity decreases at 308 and 346 nm. Besides, new absorption bands are observed at 438, 556, and 804 nm, along with an isosbestic point at 388 nm. The bands at 308 and 346 nm have been associated with the ground state of π-extended triarylamine.^28,41^ In contrast, the absorption bands at 308, 556, and 804 nm have been related to the radical cation of π-extended triarylamine.^61^ The presence of an isosbestic point indicates an equilibrium between the neutral and oxidized species, as proposed in the literature.^62,63^ These results suggest that the observed increase in *J*_SC_ in PCL FT_12_-based PSCs with UV exposure time, as shown in Figure 9 and Table 2, may be attributed to the partial oxidation of this HTL. Comparable behavior has been reported in the case of UV photoinduced oxidation observed in other triarylamine-based HTLs, where partial oxidation contributes to improved conductivity and enhancements in *J*_SC_, FF, and PCE of the devices.^31,33,34^

The hole mobilities (μ_h_) of FT_12_ and PCL FT_12_ films were determined using the space charge limitation of current (SCLC) method. Calculations were derived by fitting the *J*–*V* curves obtained from hole-only ITO/PEDOT:PSS/HTL/Au devices in the SCLC region.^23,64^ Device fabrication and calculations details can be located in the Supporting Information.

As shown in Figure 10b, the μ_h_ values for FT_12_ and PCL FT_12_ are 1.19 × 10^–4^ and 1.87 × 10^–4^ cm^2^ V^–1^ s^–1^, respectively, confirming that the photo-cross-linking process enhances the hole-transporting ability of the layer. This enhancement is likely attributed to the partial oxidation induced by UV treatment.^31,33,34^ In addition, the improvements observed in μ_h_ for PCL FT_12_ may have contributed to the enhanced charge extraction observed in the PL measurements, as well as the reduction in *R*_s_ and the increase in *J*_SC_ reported in the *J*–*V* curves of PSCs utilizing PCL FT_12_.^31,33,34^

For comparison, μ_h_ measurements were conducted on the pristine spiro layer, yielding a result of 0.36 × 10^–4^ cm^2^ V^–1^ s^–1^ (Figure S28). This value is comparable to results reported in the literature, and it is significantly lower than those reported for FT_12_ and PCL FT_12_,^65^ demonstrating that these two new materials possess superior hole-conducting properties compared to pristine spiro-OMeTAD and ruling out the sole effect of the UV radiation on the enhancement of the performance of the devices prepared with the FT_12_ derivatives.

#### 2.3.3 Stability Test of PSCs

The stability of PSCs based on FT_12_ and PCL FT_12_ was assessed under two distinct conditions. First, the efficiency measurements were performed during continuous illumination for 2 h at 25 °C in an ambient atmosphere. Subsequently, the efficiency was measured at various time intervals over one month, with the devices stored in a black box at temperatures around 20 °C and a relative humidity (RH) of 40% in a dry-air atmosphere. This long-term stability test was conducted on four devices. Additionally, PSCs based on doped spiro-OMeTAD were included for comparative purposes.

**Figure 11:**
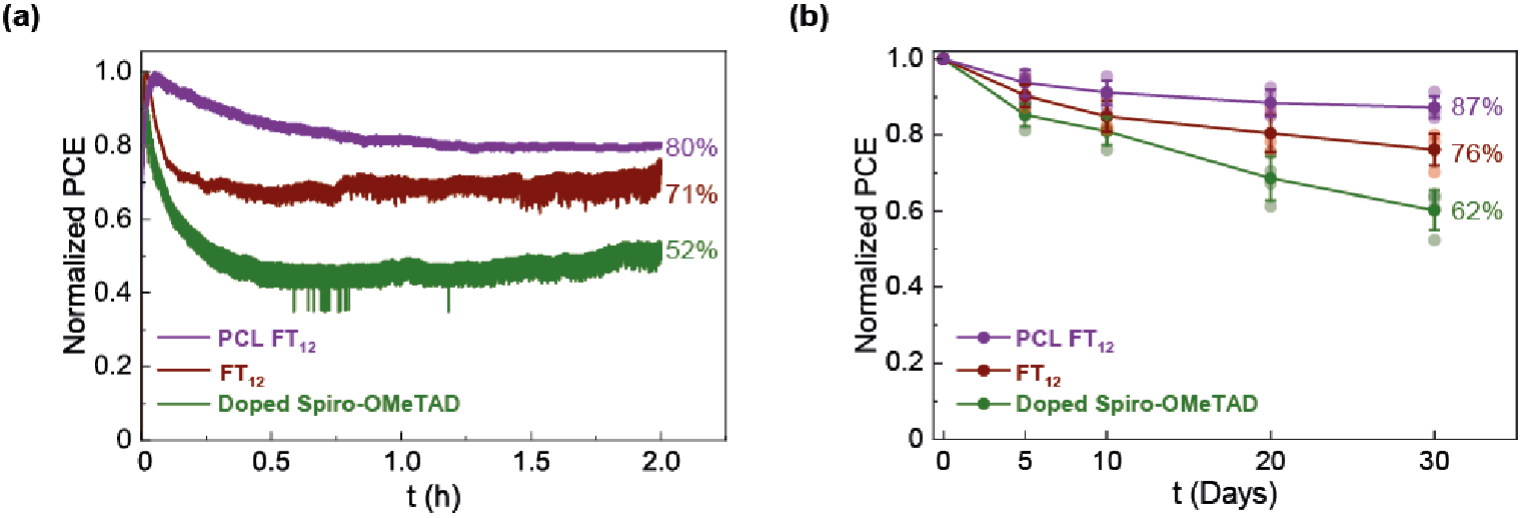
Stability test of PSCs: (a) under continuous illumination, at 25 °C in air, and (b) under dark conditions at 20 °C, 40% RH, in air.

As demonstrated in Figure 11a,b, the devices fabricated with PCL FT_12_ exhibit the highest stability, retaining approximately 80% of their initial efficiency. This observation is attributed to the increased hydrophobicity of the PCL FT_12_ surface (see Figure 5). On the other hand, the devices utilizing pristine FT_12_ maintain ∼70% of their efficiency, while those using doped spiro-OMeTAD maintain have 52% and 62% efficiency for stability under continuous illumination and long-term stability, respectively. The improved stability of the FT_12_ layer compared to spiro-OMeTAD is likely the result of a combination of factors, including the presence of ethylene glycol chains and the absence of dopants.^6,29,66−69^

**Figure 12:**
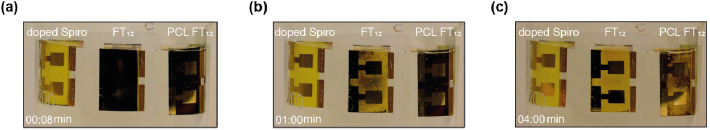
Video captures displaying the decomposition process of PSCs based on doped spiro-OMeTAD, FT_12_, and PCL FT_12_ after being immersed in water for a duration of (a) 8 s, (b) 1 min, and (c) 4 min.

The impact of PCL FT_12_ on enhancing moisture resistance was demonstrated when the device was immersed in water. For comparison, devices based on FT_12_ and doped-spiro-OMeTAD were also immersed in water (Video S3). As depicted in Figure 12, images captured during the water immersion revealed that the doped spiro-OMeTAD layer provides no protection. It promptly undergoes a color change from black to yellow, indicative of PbI_2_ formation resulting from perovskite decomposition. In contrast, the FT_12_ device provides improved water resistance. The device based on photo-cross-linked FT_12_ exhibits the highest stability, delaying complete decomposition for approximately 4 min. This highlights its superior moisture resistance compared to the others.

## 3 Conclusions

A novel cross-linkable molecule named FT_12_ was synthesized using a click reaction. Its chemical structure was confirmed by ^1^H–^13^C NMR and FTIR spectroscopy and MALDI-TOF mass spectrometry. Cyclic voltammetry analysis estimated that FT_12_ possesses HOMO and LUMO energy levels that align with the valence band of the perovskite layer and the work function of the gold contact, making it a suitable candidate for use as an HTL.

The photo-cross-linking reaction between FT_12_ and the thiol derivative PETMP was confirmed by various techniques, including FTIR spectroscopy, contact angle measurements, and UV–vis absorption spectroscopy.

Its potential as HTM has been tested in regular PSCs prepared using a C_60_-sandwich architecture, with the pristine FT_12_ and the photo-cross-linked FT_12_ employed as dopant-free HTLs. Remarkably, both FT_12_ and PCL FT_12_ demonstrated superior performance compared to pristine spiro-OMeTAD. Photovoltaic parameters revealed that devices employing PCL FT_12_ achieved higher PCE than those employing FT_12_.

Morphological studies revealed that the PCL FT_12_ layer is characterized by more excellent uniformity and smoother surface than the FT_12_ layer. Furthermore, the utilization of the photo-cross-linking technique resulted in enhanced charge extraction and hole mobility. These factors are likely contributors to the improved PCE.

Through UV–vis spectroscopy analysis of films from the photo-cross-linked reaction mixture exposed for different time intervals, it has been demonstrated that the photoinduced oxidation of the π-extender triarylamine occurs due to UV irradiation. This phenomenon may account for the increased hole mobility observed in PCL FT_12_, together with the improved morphology of the film.

Significantly improved stability of PSCs is observed when incorporating the photo-cross-linked FT_12_ layer compared to the use of the doped spiro-OMeTAD film. This enhanced stability is attributed to the moisture-resistant surface of the PCL FT_12_ layer. Devices utilizing pristine FT_12_ also demonstrate superior strength when compared to those employing doped spiro-OMeTAD, likely owing to the integration of tetramethylene glycol chains into the chemical structure of FT_12_.

The photo-cross-linking technique was successfully introduced in this study for preparing HTLs, demonstrating the critical role of the appropriate UV-irradiation time and precursor quantities in enhancing the efficiency and stability of PSCs simultaneously.

## 4 Experimental Section

### 4.1 Synthesis of FT_12_

FT_12_:T-Azide (496 mg, 0.32 mmol) and F-Akyne_12_ (53 mg, 0.021 mmol) were dissolved in dichloromethane (3 mL), and distilled water (0.5 mL) was added. Subsequently, under an argon atmosphere, sodium ascorbate (NaAsc, 190 mg, 0.96 mmol, in 0.5 mL of H_2_O) and CuSO_4_·5H_2_O (24 mg, 0.096 mmol, in 0.5 mL of H_2_O) were introduced. The resulting heterogeneous mixture was degassed and vigorously stirred for 96 h at room temperature. The residue was then purified by centrifugation, utilizing dichloromethane: H_2_O and EtOAc:H_2_O, followed by drying the organic layer over Na_2_SO_4_, ultimately yielding FT_12_ as a yellow-brown solid product (409 mg, 0.019 mmol, 91%).

^1^H NMR (500 MHz, benzene-*d*_6_) δ [ppm] = 7.52 (d, *J* = 9.0 Hz, 4H), 7.31 (s, 2H), 7.20–7.28 (m, 4H), 7.16–7.09 (m, 32H), 6.83 (d, *J* = 9.0 Hz, 16H), 5.95–5.78 (m, 8H), 5.27 (dd, *J* = 17.2, 1.9 Hz, 8H), 5.06 (dd, *J* = 10.4, 2.0 Hz, 8H), 4.34 (t, *J* = 6.1 Hz, 4H), 3.88–3.83 (m, 32H), 3.63–3.60 (m, 16H), 3.51–3.57 (m, 80H), 3.49–3.45 (m, 16H), 2.74 (t, *J* = 7.2 Hz, 4H), 1.77–1.68 (m, 4H), 1.63 (d, *J* = 7.3 Hz, 4H), 1.42 (d, *J* = 8.1 Hz, 4H). ^13^C NMR (126 MHz, benzene-*d*_6_) δ [ppm] = 163.7, 155.7, 148.8, 148.2, 146.0, 144.1, 141.6, 140.7, 139.5, 135.7, 131.0, 126.7, 126.6, 122.6, 121.6, 121.5, 118.8, 116.1, 115.9, 72.4, 72.2, 71.17, 71.1, 70.1, 70.0, 68.0, 67.6, 45.3, 29.2, 28.7, 26.1, 26.0. MS (MALDI-TOF) = *m*/*z* calculated for [C_1195_H_1480_N_72_O_264_]^+^: 21062.46; found: 21062.55.

### 4.2 Photo-Cross-Linking Procedure

The photo-cross-linking process was carried out using a Dymax UVC-5 Light-Curing Conveyor, which operates at a wavelength of 365 nm with a power of 800 W. The films, deposited on aluminum disks, glass, and solar cell substrates, were positioned 10 cm from the lamp.

The photo-cross-linked films on glass and perovskite layer were prepared using freshly prepared solutions, comprising a mixture of 11 mg/mL of FT_12_, one equivalent of pentaerythritol tetrakis(3-mercaptopropionate) (PETMP), and 2-hydroxy-2-methylpropiophenone (HMP) (1% w/w relative to FT_12_) in chlorobenzene. The amount of HMP employed adhered to previously reported recommendations.^54^ The mixture was subsequently subjected to argon degassing, deposition by spin-coating at 4000 rpm for 20 s, drying at 100 °C for 10 min, UV-curing for 20 s, and dynamic rinsing with toluene at 4000 rpm for 60 s.^38,70^ In the case of the films on aluminum disks, the protocol was the same, but with an excess of PETMP included to detect its absorption in the FTIR measurements. The deposition was performed through drop-casting without rinsing.

### 4.3 Fabrication of Perovskite Solar Cells

Prepatterned ITO glass substrates were subjected to a sequential cleaning and ultrasonication process, which involved a 15 min treatment with detergent, distilled water, ethanol, and isopropanol. Subsequently, the substrates were exposed to UV-ozone for 30 min before being transferred into a glovebox with an N_2_ atmosphere.

To prepare the C_60_ precursor solution, 20 mg/mL was dissolved in dry *o*-DCB and ultrasonicated at temperatures below 20 °C.^49,50^ The solution was filtered through a 0.45 μm poly(tetrafluoroethylene) filter before use. The freshly prepared solution was deposited onto ITO substrates by spin coating at 3000 rpm for 30 s, followed by preannealing at 60 °C for 30 min and subsequent drying under vacuum conditions at 100 °C for 1 h.^43^

The precursor solution of MAPI, containing a 1:1 molar ratio of MAI to PbI_2_, was prepared from a mixture of dry DMF and DMSO (7:3) at a concentration of 1.4 M.^51^ This solution was continuously stirred at 70 °C overnight.^52^ The solution was filtered with a 0.20 μm poly(tetrafluoroethylene) filter before usage. The fully dissolved solution was then spin-coated onto the C_60_ layer, with 1000 and 4000 rpm rotations for 10 and 45 s, respectively. During spinning, 200 μL of CB was added to the film’s center 5 s before the end of the procedure. The resulting light-yellow film was heated at 65 °C for 1 min, followed by 30 min at 100 °C to obtain a shiny black film.^53^

The PCL FT_12_ layer was prepared using a precursor solution at 11 mg/mL concentration in anhydrous CB. It was then deposited onto the perovskite layer at 4000 rpm for 20 s and dried at 100 °C for 10 min. To optimize the PETMP concentration, mixtures of FT_12_ at 11 mg/mL in anhydrous CB with varying PETMP concentrations were spin-coated onto the perovskite layer and dried at 100 °C for 10 min. The PCL FT_12_ layer was prepared as described in the “Photo-cross-linking Procedure” section above. For the pristine spiro-OMeTAD layer, a solution with a concentration of 70 mM in anhydrous CB was dynamically deposited at 4000 rpm for 20 s. Similarly, the doped spiro-OMeTAD layer was prepared using the same attention and doping procedure, incorporating *t*-BP (3.3 equiv), Li-TFSI (0.5 equiv), and FK209 (0.05 equiv), as detailed in the literature.^23^

Finally, a 100 nm gold electrode was deposited using a thermal evaporator, employing a shadow mask with an active area of 0.16 cm^2^.

## Supporting Information Available

Materials and characterization methods used to synthesize and fabricate perovskite solar cells (PSCs) and supporting figures and tables for the characterization of the synthesized compounds and description of PSCs (PDF)
The movement of a water droplet across the surface of PCL FT_12_ is visualized in Supporting Movie S1 (MP4)
Across the surface of the photo-cross-linking reaction mixture in Supporting Movie S2 (MP4)
PSCs immersed in water in Supporting Movie S3 (MP4)

## Abbreviations

ETL: electron transport layer
ETM: electron transport material
FF: fill factor
HMP: 2-hydroxy-2-methylpropiophenone
HTL: hole transport layer
HTM: hole transport material
MAPI: methylammonium lead iodide
PCE: power conversion efficiency
PCL: photo-cross-linked
PETMP: pentaerythritol tetrakis(3-mercaptopropionate)
PSC: perovskite solar cell
