# Supplementary material for: Photo-Cross-Linked Fullerene-Based Hole Transport Material for Moisture-Resistant Regular Fullerene Sandwich Perovskite Solar Cells
Source: ACS Appl Mater Interfaces. 2024 Apr 15;16(16):20852–64. doi: 10.1021/acsami.4c02573 (PMC11056936; doi:10.1021/acsami.4c02573)
Supplement: Supplementary file 1 — am4c02573_si_001.pdf [file am4c02573_si_001.pdf]

Supporting Information

Photo-Crosslinked Fullerene-Based Hole Transport Material for Moisture-Resistant Regular Fullerene Sandwich Perovskite Solar Cells

Andrea Cabrera-Espinoza[1], Silvia Collavini[1], José G. Sánchez[2], Ivet Kosta[3], Emilio Palomares[2,4*], Juan Luis Delgado[1,5*]

[1] *POLYMAT, University of the Basque Country UPV/EHU, Avenida Tolosa 72, Donostia/San Sebastián 20018, Spain*

[2] *Institute of Chemical Research of Catalonia-The Barcelona Institute of Science and Technology (ICIQ-BIST), Avinguda Països Catalans 16, Tarragona 43007, Spain*

[3] *CIDETEC, Basque Research and Technology Alliance (BRTA), Paseo Miramón 196, Donostia/San Sebastián 20014, Spain*

[4] *ICREA, Passeig Lluís Companys 23, Barcelona 08010, Spain*

[5] *Ikerbasque, Basque Foundation for Science, Bilbao 48013, Spain*

*Juan Luis Delgado: juanluis.delgado@polymat.eu
*Emilio Palomares: epalomares@iciq.es

TABLE OF CONTENTS

1. Supplementary experimental section S2
 1.1. Synthesis S2
  1.1.1. Materials S2
  1.1.2. Structural characterization methods....S2
  1.1.3. Synthesis of F-Alkyne$_{12}$ S2
  1.1.4. Synthesis of T-Azide....S3
 1.2. Perovskite solar cells....S5
  1.2.1. Materials S5
  1.2.2. Device characterization methods S5
2. Supporting figures and tables....S6
 2.1. Characterization of the synthesized compounds S6
 2.2. Characterization of perovskite solar cells S17
References S20

1. SUPPLEMENTARY EXPERIMENTAL SECTION

1.1. Synthesis

1.1.1. Materials

Chemicals and reagents for synthesis were purchased from commercial suppliers and used as received. Air-sensitive reactions were carried out under an argon atmosphere. Anhydrous solvents were dried using an SPS purification system. Chromatography was performed using silica gel (40-60 μ, Acros Organics). Analytical thin-layer chromatography (TLC) was performed using aluminum-coated Macherey Nagel™ Standard SIL G Silica Layers on Alugram™ Aluminum Sheets UV254.

1.1.2. Structural characterization methods

The nuclear magnetic resonance (NMR) spectra were recorded on a Bruker Advance 300 and 500 spectrometers (^{1}H: 300 MHz; ^{13}C: 75 MHz and ^{1}H: 500 MHz; ^{13}C: 126 MHz, respectively). These measurements were conducted at a temperature of 25 ºC for intermediates and precursors and at 70 ºC for FT_{12}.[1] The spectra were subsequently referenced to deuterated solvents (e.g., CH_2Cl_2-d_2 to 5.32 and 54.00 ppm; DMSO-d_6 to 2.50 and 39.52 ppm; acetone-d_6 to 2.09 and 205.87 ppm; and benzene-d_6 to 7.15 and 128.62 ppm for ^{1}H and ^{13}C NMR spectra, respectively). Chemical shifts (δ) are reported in ppm. Multiplicities are denoted as follows: s (singlet), d (doublet), t (triplet), dd (doublet of doublets), dt (doublet of triplets), ddt (doublet of triplets), m (multiplet), and the coupling constants (*J*) are given in Hz.

Matrix-assisted laser desorption/ionization time-of-flight (MALDI-TOF) mass experiments were performed on a Bruker Ultraflex III. All data were acquired at a maximum accelerating potential of 20 kV in the linear negative ion mode. 1,8-Dihydroxy-9,10-dihydroanthracen-9-one was used as the matrix.

Fourier transforms infrared (FTIR) spectra were acquired with a Bruker ALPHA ATR-IR spectrometer, either on the pure sample or on films deposited onto aluminum substrates.

Cyclic voltammetry was carried out on a Princeton Applied Research Parstat 2273 in a custom-made glass cell using a three-electrode setup with a glassy carbon disk working electrode (Ø = 3 mm, CH Instruments, CHI104), a platinum wire counter electrode (Ø = 0.5 mm, CH Instruments, CHI115), and a silver wire pseudo-reference electrode (Ø = 0.5 mm, CH Instruments, CHI112). All experiments were conducted using a degassed solution of *o*-DCB:AcN (4:1) containing 0.1 M tetrabutylammonium hexafluorophosphate (Bu_4NPF_6) as the supporting electrolyte, and 0.5 mM of sublimated decamethylferrocene was added as an internal reference (DMFc/DMFc$^+$ = -0.556 V).[2] 1 mM of FT_{12} was added to the system for cathodic scanning, and 0.07 mM for anodic scanning. The experiments were carried out at a scan rate of 100 mV s^{-1}.

Contact angles were determined using the drop technique and an optical tensiometer (Dataphysics Contact Angle System OCA) under ambient conditions. Application of 10 µL water droplets was carried out, and contact angle values were subsequently averaged from multiple measurements obtained at distinct positions on each surface.

UV-Vis absorption spectra were obtained using a Perkin-Elmer Lambda 950 spectrometer. The spectra were recorded in film at room temperature.

1.1.3. Synthesis of F-Alkyne$_{12}$

Scheme S1 illustrates the preparation of the hexakis-adduct F-Alkyne$_{12}$. Malonate 2 was obtained in an 82% yield through the esterification of alcohol 1 with malonyl chloride in a basic triethylamine-based medium. The desired F-Alkyne$_{12}$ was subsequently produced in a 52% yield by treating C_{60} with an excess of 2 in the presence of the CBr_4/DBU system at room temperature, following the modified Bingel-Hirsch reaction conditions proposed by H. Li *et al*.[3]

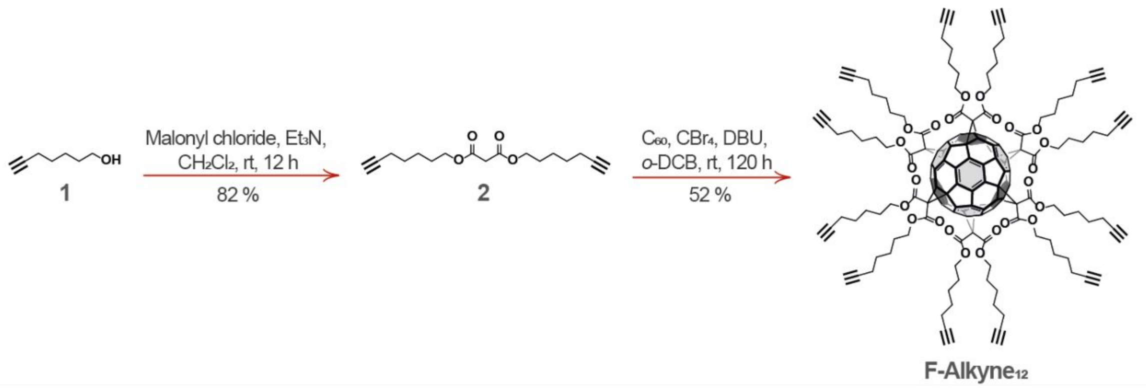

Scheme S1. Synthesis of F-Alkyne$_{12}$.

Di(hept-6-yn-1-yl) malonate (2): Malonyl chloride (0.97 mL, 10 mmol) was added slowly to a stirred, ice-cold solution of 6-heptyn-1-ol (2.51 mL, 20 mmol) and dry pyridine (1.62 mL, 20 mmol) in dry dichloromethane (125 mL) under an argon atmosphere. The reaction was allowed to warm up to room temperature and left stirring overnight. Subsequently, the reaction mixture was filtered, and the product was purified through column chromatography with dichloromethane, resulting in the isolation of 2 as a yellowish oil (2.40 g, 8.2 mmol, 82% yield).

^{1}H NMR (300 MHz, acetone-d_6) δ [ppm] = 4.12 (t, J = 6.6 Hz, 4H), 3.40 (s, 2H), 2.30 (t, J = 2.7 Hz, 2H), 2.19 (dt, J = 6.6, 2.7 Hz, 4H), 1.76 – 1.64 (m, 4H), 1.64 – 1.42 (m, 8H). ^{13}C NMR (75 MHz, acetone-d_6) δ [ppm] = 167.2, 84.7, 69.9, 65.5, 42.0, 28.8, 28.7, 25.6, 18.6.

F-Alkyne$_{12}$: CBr_4 (6.63 g, 20 mmol), 2 (585 mg, 2 mmol), and DBU (609 mg, 4 mmol) were successively added to a solution of C_{60} (144 mg, 0.20 mmol) in dry toluene (50 mL) at room temperature. Following a 120-hour stirring period, the mixture was subjected to evaporation. Subsequently, the residue was dissolved in dichloromethane, undergoing successive washing with a saturated solution of $Na_2S_2O_3$, brine, and water. The organic layer was then dried over Na2SO4 and concentrated under vacuum. The crude product was purified through column chromatography, employing a toluene:ethyl acetate mixture (9:1) as the eluent, ultimately yielding F-Alkyne$_{12}$ as a pale yellow glassy product (256 mg, 0.10 mmol, 52% yield).

^{1}H NMR (500 MHz, CH_2Cl_2-d_2) δ [ppm] = 4.49 (t, J = 6.6 Hz, 4H), 2.28 – 2.18 (m, 4H), 1.99 (t, J = 2.8 Hz, 1H), 1.93 – 1.81 (m, 4H), 1.70 – 1.54 (m, 8H). ^{13}C NMR (126 MHz, acetone-d_6) δ [ppm] = 164.1, 146.5, 142.3, 84.8, 81.0, 70.1, 67.7, 47.0, 28.9, 25.9, 20.0, 18.7. MS (MALDI-TOF) = m/z calculated for $[C_{162}H_{132}O_{24}]^{+}$: 2460.95; found: 2460.91.

The NMR spectra are presented in Figure S1 to Figure S4.

1.1.4. Synthesis of T-Azide

The synthetic route for compound T-Azide is depicted in Scheme S2. *tert*-Butyl(4-bromophenyl)carbamate (*N*-Boc-protected-4-bromoaniline), *tert*-butyl(4-iodophenoxy)dimethylsilane (*O*-silyl-protected-4-iodoaniline) and 4-toluenesulfonyl tetraethylene glycol allyl ether (tosylated-tetraethyleneglycol allyl ether) were synthesized using established methods as previously reported.[4–6]

The Buchwald–Hartwig amination process, using the catalytic system tris(dibenzylideneacetone)dipalladium(0) / tri-*tert*-butylphosphine ($Pd_2(dba)_3$/P(*t*-Bu)$_3$) in the presence of sodium *tert*-butoxide (NaO*t*-Bu), was employed on two occasions. Firstly, it was utilized to prepare triarylamine 4 through the reaction between 4-nitroaniline and *N*-Boc-protected-4-bromoaniline. Subsequently, compound 4 was deprotected by the addition of trifluoroacetic acid (TFA) to yield diamine 5. On the second occasion, Buchwald-Hartwig amination was employed to synthesize the π-extended triarylamine 6 from compound 5 and *O*-silyl-protected-4-iodoaniline.

Deprotection of 6 with tetrabutylammonium fluoride (TBAF) afforded 7 in quantitative yield. Introduction of allyl cross-linking groups was performed by *O*-alkylation of compound 7 with tosylated-tetraethyleneglycol allyl ether, resulting in the formation of compound 8 in 82% yield.

Selective reduction of the nitro group in compound 8 to an amine group was achieved through treatment with stannous chloride. Subsequently, the preparation of T-Azide was carried out using 2-azido-1,3-dimethylimidazolinium chloride (ADMC) as the diazo-transfer reagent and a basic medium consisting of 4-dimethylaminopyridine (DMAP), following a protocol established in the literature.[7]

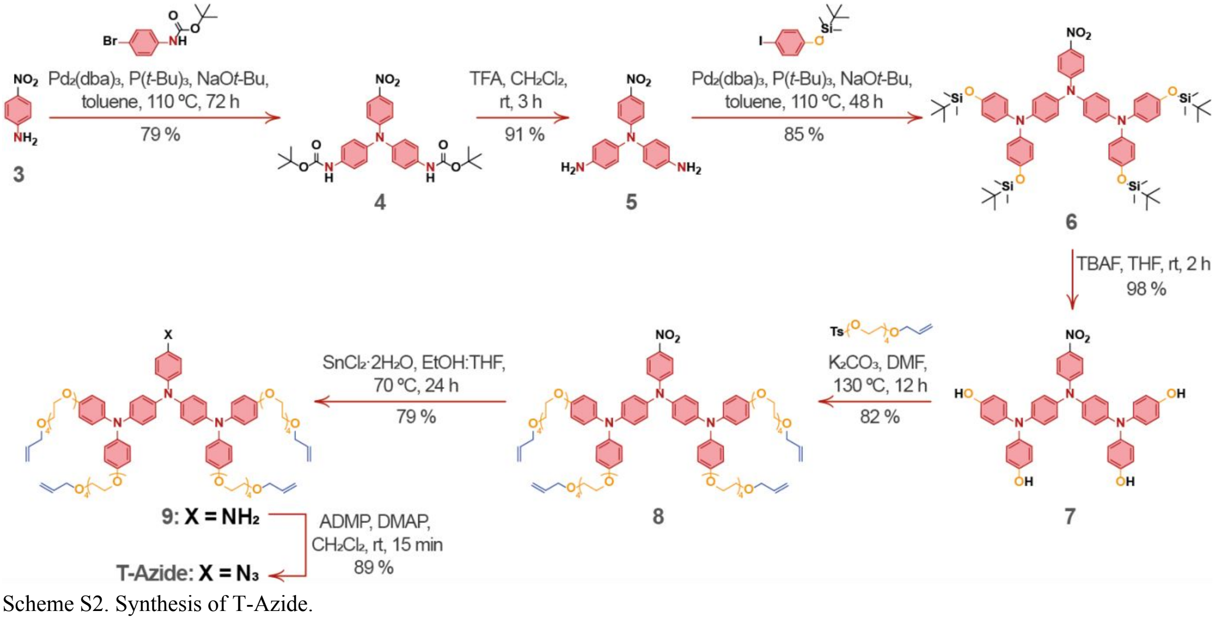

Scheme S2. Synthesis of T-Azide.

4,4'-Di-*tert*-butoxycarbonylamino-4''-nitrotriphenylamine (4): In a solution of *N*-Boc-protected-4-bromoaniline (1.63 g, 6.0 mmol) and 4-nitroaniline (207 mg, 1.5 mmol) in 30 mL of degassed, dry toluene, $Pd_2(dba)_3$ (137 mg, 0.15 mmol), P(*t*-Bu)$_3$ (1 M in toluene, 230 μL, 0.23 mmol), and NaO*t*-Bu (432 mg, 4.5 mmol) were added under an argon atmosphere. After 15 minutes of argon degassing, the mixture was stirred for 1 hour at room temperature and then refluxed for 72 hours. Upon cooling to room temperature, the solvent was removed under reduced pressure. Subsequent purification via column chromatography with hexane:ethyl acetate (2:1) eluent resulted in the isolation of 4 as an orange solid (619 mg, 1.19 mmol, 79% yield).

^{1}H NMR (300 MHz, acetone-d_6) δ [ppm] = 8.57 (s, 2H), 8.04 (d, *J* = 9.3 Hz, 2H), 7.64 (d, *J* = 8.8 Hz, 4H), 7.22 (d, *J* = 8.8 Hz, 4H), 6.83 (d, *J* = 9.3 Hz, 2H), 1.49 (s, 18H). ^{13}C NMR (75 MHz, acetone-d_6) δ [ppm] = 155.0, 153.7, 140.5, 139.9, 138.9, 128.4, 126.2, 120.5, 116.8, 80.2, 28.5.

4,4'-Diamino-4''-nitrotriphenylamine (5): To a solution of 4 (468 mg, 0.9 mmol) in 4 mL of dry dichloromethane, was added TFA (1.03 mL, 9.0 mmol) under an argon atmosphere. Following a 3-hour stirring period at room temperature, neutralization was achieved using a cooled saturated solution of Na_2CO_3. The organic layer was subsequently separated, washed with water, and dried over Na_2SO_4. After vacuum removal of the solvent, the residue was purified by column chromatography, eluting with dichloromethane. This process yielded 5 as a yellow solid (262 mg, 0.8 mmol, 91% yield).

^{1}H NMR (300 MHz, acetone-d_6) δ [ppm] = 7.97 (d, *J* = 9.4 Hz, 2H), 7.01 (d, *J* = 8.6 Hz, 4H), 6.73 (d, *J* = 8.6 Hz, 4H), 6.65 (d, *J* = 9.4 Hz, 2H), 4.81 (s, 4H). ^{13}C NMR (126 MHz, acetone-d_6) δ [ppm] = 156.1, 148.1, 138.5, 135.5, 129.1, 126.2, 116.2, 114.8.

4,4'-Bis(4,4′-di(*tert*-butyldimethylsiloxy)diphenylaminyl)-4''-nitrotriphenylamine (6): To a solution of *O*-silyl-protected-4-iodoaniline (1.34 g, 4.0 mmol), 5 (256.3 mg, 0.80 mmol) in degassed dry toluene (16 mL) $Pd_2(dba)_3$ (73 mg, 0.08 mmol), P(*t*-Bu)$_3$ (1 M in toluene, 120 μL, 0.12 mmol) and NaO*t*-Bu (461.3 mg, 4.8 mmol) were added under argon. Following 15 minutes of argon degassing, the mixture was stirred for 1 hour at room temperature and then refluxed for 48 hours. After cooling to room temperature, the solvent was removed under reduced pressure. Subsequent purification by column chromatography using a hexane:dichloromethane mixture (3:1) resulted in the isolation of 6 as an orange solid (778 mg, 0.68 mmol, 85% yield).

^{1}H NMR (300 MHz, acetone-d_6) δ [ppm] = 8.00 (d, *J* = 9.4 Hz, 2H), 7.11 (d, *J* = 8.9 Hz, 4H), 7.03 (d, *J* = 8.9 Hz, 8H), 6.90 (d, *J* = 8.9 Hz, 4H), 6.86 – 6.80 (m, 10H), 1.00 (s, 36H), 0.22 (s, 24H). ^{13}C NMR (75 MHz, acetone-d_6) δ [ppm] = 155.2, 153.0, 148.0, 142.0, 139.6, 138.4, 128.7, 127.6, 126.2, 122.0, 121.7, 116.3, 26.0, 18.8, -4.29.

4,4'-Bis(4,4'-dihydroxydiphenylaminyl)-4''-nitrotriphenylamine (7): At room temperature, TBAF (1 M in THF, 4 mL, 4.0 mmol) was introduced into a solution of 6 (573 mg, 0.50 mmol) in dry THF (5 mL). After 2 hours, a saturated solution of NH_4Cl was added. The organic layer was subsequently subjected to washing with water, drying over Na_2SO_4, and vacuum concentration. The product was purified through column chromatography, utilizing a $CHCl_3$:acetone (3:1) eluent, resulting in the isolation of 7 as a red solid (337 mg, 0.49 mmol, 98% yield).

^{1}H NMR (300 MHz, acetone-d_6) δ [ppm] = 8.33 (s, 4H), 8.00 (d, *J* = 9.4 Hz, 2H), 7.09 (d, *J* = 8.9 Hz, 4H), 7.02 (d, *J* = 8.9 Hz, 8H), 6.87 – 6.80 (m, 12H), 6.78 (d, *J* = 9.4 Hz, 2H). ^{13}C NMR (75 MHz, acetone-d_6) δ [ppm] = 155.5, 155.2, 148.8, 148.7, 140.3, 137.5, 128.7, 128.3, 126.2, 120.4, 117.1, 115.8.

4,4'-Bis(4,4′-di((3,6,9,12-tetraoxapentadec-14-en-1-yl)oxy)diphenylaminyl)-4''-nitrotriphenylamine (8): Under an argon atmosphere, a suspension of K_2CO_3 (2.21 g, 16 mmol), 7 (689 mg, 1 mmol), and tosylated-tetraethyleneglycol diallyl ether (1.69 g, 5 mmol) in DMF (10 mL) was stirred at 130 °C for 12 hours. Upon cooling to room temperature, the reaction mixture was poured into water, extracted with EtOAc and dried over Na_2SO_4. Subsequent concentration of the organic layer, followed by purification through column chromatography with EtOAc, resulted in the isolation of 8 as an orange solid (1.27 g, 0.82 mmol, 82% yield).

^{1}H NMR (300 MHz, acetone-d_6) δ [ppm] = 8.01 (d, *J* = 9.4 Hz, 2H), 7.12 (d, *J* = 8.9 Hz, 4H), 7.09 (d, *J* = 9.0 Hz, 8H), 6.94 (d, *J* = 9.0 Hz, 8H), 6.89 (d, *J* = 8.9 Hz, 4H), 6.82 (d, *J* = 9.4 Hz, 2H), 5.89 (ddt, *J* = 17.2, 10.6, 5.3 Hz, 4H), 5.30 – 5.20 (m, 4H), 5.13 – 5.06 (m, 4H), 4.14 – 4.10 (m, 8H), 4.00 – 3.95 (m, 8), 3.83 – 3.79 (m, 8H), 3.72 – 3.45 (m, 48H). ^{13}C NMR (75 MHz, acetone-d_6) δ [ppm] = 156.7, 155.3, 148.1, 141.4, 139.5, 138.2, 136.4, 128.7, 127.8, 126.2, 121.5, 116.5, 116.3, 116.2, 73.5, 72.4, 71.4, 71.31, 71.25, 71.1, 70.4, 68.7, 62.0.

4-Amino-4',4''-Bis(4,4′-di((3,6,9,12-tetraoxapentadec-14-en-1-yl)oxy)diphenylaminyl)-triphenylamine (9): To a solution of 8 (777 mg, 0.5 mmol) in THF (10 mL) was added $SnCl_2 \cdot 3H_2O$ (1.13 g, 5.0 mmol) in EtOH (10 mL). The mixture was heated at 70 ºC for 24 h under an argon atmosphere. The solvent was removed under reduced pressure and the residue was neutralized slowly with a cooled saturated solution of Na_2CO_3. Following this, EtOAc was added and the organic layer was washed with water and dried over Na_2SO_4. After the solvent was removed, orange solid 9 was obtained (609 mg, 0.40 mmol, 79% yield).

^{1}H NMR (300 MHz, DMSO-d_6) δ [ppm] = 6.97 – 6.71 (m, 26H), 6.58 (d, *J* = 8.6 Hz, 2H), 5.85 (ddt, *J* = 17.2, 10.6, 5.3 Hz, 4H), 5.26 – 5.18 (m, 4H), 5.14 – 5.07 (m, 4H), 4.98 (s, 2H), 4.05 – 4.01 (m, 8H), 3.92 (dt, *J* = 5.3, 1.5 Hz, 8H), 3.73 – 3.69 (m, 8H), 3.58 – 3.46 (m, 48H). ^{13}C NMR (75 MHz, DMSO-d_6) δ [ppm] = 154.1, 145.8, 142.4, 141.7, 141.0, 135.6, 135.3, 127.4, 125.0, 122.9, 122.4, 116.3, 115.4, 114.9, 71.0, 69.9, 69.82, 69.76, 69.01, 68.98, 67.3.

4-Azido-4',4''-Bis(4,4′-di((3,6,9,12-tetraoxapentadec-14-en-1-yl)oxy)diphenylaminyl)-triphenylamine (T-Azide): In a dry dichloromethane solution (2.5 mL) containing 9 (609 mg, 0.4 mmol) and DMAP (73 mg, 0.6 mmol) at room temperature, ADMP (171 mg, 0.6 mmol) was added under an argon atmosphere. After 15 minutes, the solvent was removed under vacuum to afford the crude compound, which was purified by column chromatography with EtOAc:acetone (9:1) to give T-Azide as an orange solid (552 mg, 0.36 mmol, 89% yield).

^{1}H NMR (300 MHz, DMSO-d_6) δ [ppm] = 7.01 – 6.84 (m, 24H), 6.79 – 6.73 (m, 4H), 5.85 (ddt, J = 17.3, 10.6, 5.3 Hz, 4H), 5.26 – 5.18 (m, 4H), 5.16 – 5.06 (m, 4H), 4.06 – 4.02 (m, 8H), 3.92 (dt, J = 5.3, 1.5 Hz, 8H), 3.74 – 3.69 (m, 8H), 3.59 – 3.46 (m, 48H). ^{13}C NMR (75 MHz, DMSO-d_6) δ [ppm] = 154.6, 145.3, 144.0, 140.5, 140.1, 135.3, 131.6, 125.9, 125.3, 122.6, 121.5, 120.1, 116.3, 115.5, 71.0, 69.9, 69.83, 69.81, 69.76, 69.01, 68.96, 67.3. HR-MS (MALDI-TOF) = *m/z* calculated for $[C_{86}H_{112}N_6O_{20}]^+$: 1548.79; found: 1548.77.

The NMR spectra are presented in Figure S5 to Figure S18.

1.2. Perovskite solar cells

1.2.1. Materials

The materials used in the photovoltaic study were obtained from commercial suppliers in high purity and used without further purification. [60]Fullerene (C_{60}, 98%, SES Research), methylammonium iodide (MAI, >99.9%, Greatcell Solar Materials), lead(II) iodide (PbI_2, 99.9%, TCI chemicals), 2,2',7,7'-tetrakis[N,N-di(4-methoxyphenyl)amino]-9,9'-spirobifluorene (spiro-OMeTAD, >99.0 %, Shenzhen Furui Technology Co.,Ltd), bis(trifluoromethane)sulfonimide lithium salt (Li-TFSI, 99.9%, Sigma-Aldrich), tris(2-(1H-pyrazol-1-yl)-4-tert-butylpyridine)cobalt(II) di[bis(trifluoromethane)sulfonimide] (FK209, 98%, Sigma-Aldrich), 4-*tert*-butylpyridine (*t*-BP, 98%, Sigma-Aldrich), poly(3,4-ethylenedioxythiophene) poly(3,4-ethylenedioxythiophene) and polystyrene sulfonate dispersion (PEDOT:PSS dispersion, Heraeous Clevios™ P VP AI 4083), gold wire (Au, 99.99%, diameter 0.5 mm, Kurt J. Lesker), dimethylformamide (DMF extra dry, Acros Organics), dimethyl sulfoxide (DMSO, extra dry, Acros Organics), chlorobenzene (CB extra dry, Acros Organics), *o*-dichlorobenzene (*o*-DCB anhydrous, 99.8% in Sure/Seal, Sigma Aldrich). Pre-patterned ITO glass substrates (Nippon sheet glass, 1.4 x 2.5 cm, 10 Ω sq^{-1}) are purchased from Xop Física S. L.

1.2.2. Device characterization methods

Current density-voltage (*J-V*) measurements were conducted under standard conditions, which involved an intensity equivalent to 1 sun (100 mW cm^{-2}, AM 1.5G, 25 °C). These measurements were carried out utilizing a Keithley 2400 source meter and an ABET Technologies solar simulator (model 11000, class type A). The scan rate applied was 100 mV s^{-1} under ambient atmospheric conditions. The procedure included an initial short-circuit preconditioning, immediately followed by a forward scan. The solar simulator was calibrated with a monocrystalline silicon with a standard quartz window (RR-1002/RQN7622 without filter, Rera Solutions).

External quantum efficiency (EQE) measurements were conducted using a standard system consisting of a xenon lamp (Oriel 66902), a monochromator (Newport 74085), a Si detector (Oriel 76175), and an amplifier from Stanford Research Systems SR570. Data were acquired across a wavelength range spanning from 280 to 850 nm.

The morphologies of the substrates were analyzed with an ULTRA plus ZEISS field-emission scanning electron microscope. Atomic force microscopy (AFM) measurements were conducted utilizing a Bruker Dimension ICON in tapping mode, employing TEST-V2 type tips with a resonance frequency of 320 kHz and a spring constant of 37 N/m.

Steady-state photoluminescence (PL) and time-resolved photoluminescence (TRPL) measurements were carried out using a fluorescence lifetime spectrometer (LifeSpec II, Edinburgh Instruments) equipped with a photomultiplier tube (PMT) detector and a double subtractive monochromator. The system employed a picosecond pulsed diode laser (EPL-635, Edinburgh Instruments) with a wavelength of 635 ± 10 nm. All measurements were conducted under ambient conditions with freshly prepared samples. The glass/MAPI structure was employed to assess the response of the MAPI layer, while the glass/MAPI/HTL (FT_{12} and PCL FT_{12}) structures were utilized to evaluate the interaction between the MAPI layer and HTL. The preparation procedures for both the MAPI layer and HTLs were identical to those used in the preparation of PSCs, an additional layer of PMMA was added on top to prevent degradation by moisture during the measurements.

Hole mobility (μ_h) was determined using the space-charge limited current (SCLC) method in hole-only devices with ITO/PEDOP:PSS/HTLs/Au structures. The devices were prepared as reported in the literature,[8] and the FT_{12} and PCL FT_{12} layers were prepared as the procedure used to prepare PSCs. The thickness of the films was measured with a stylus profilometer Ambios Tech. XP-1, from a scratch made in the middle of the film. The μ_h values were calculated by fitting the current-voltage (*J-V*) curves with the equation $J = 9\varepsilon_0\varepsilon_r\mu_e V^2/8L$ (in the SCLC regimen), where J is the current density, L is the thickness of the HTL, ε_r is the relative dielectric constant of the transport medium, ε_0 is the permittivity of vacuum and V is the applied voltage.

2. SUPPORTING FIGURES AND TABLES

2.1. Characterization of the synthesized compounds

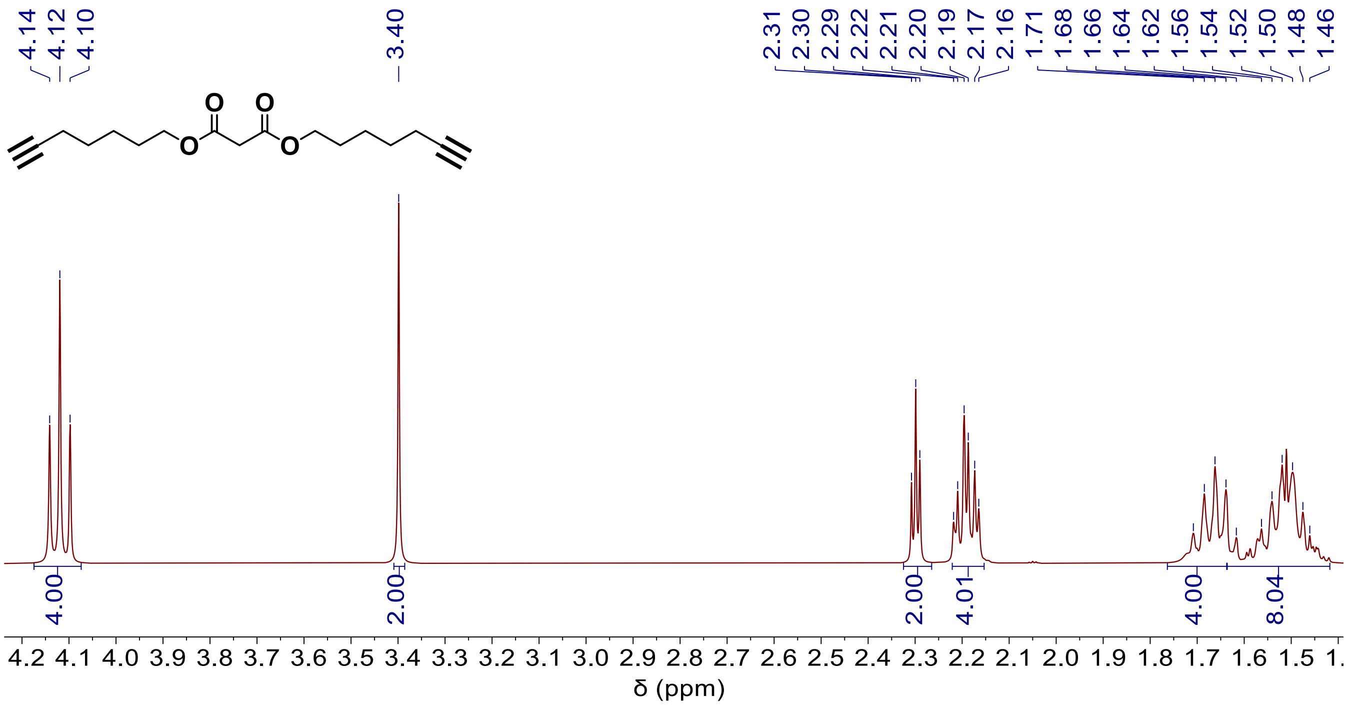

Figure S1. ^{1}H NMR spectrum of 2 in acetone-d_6 at 300 MHz.

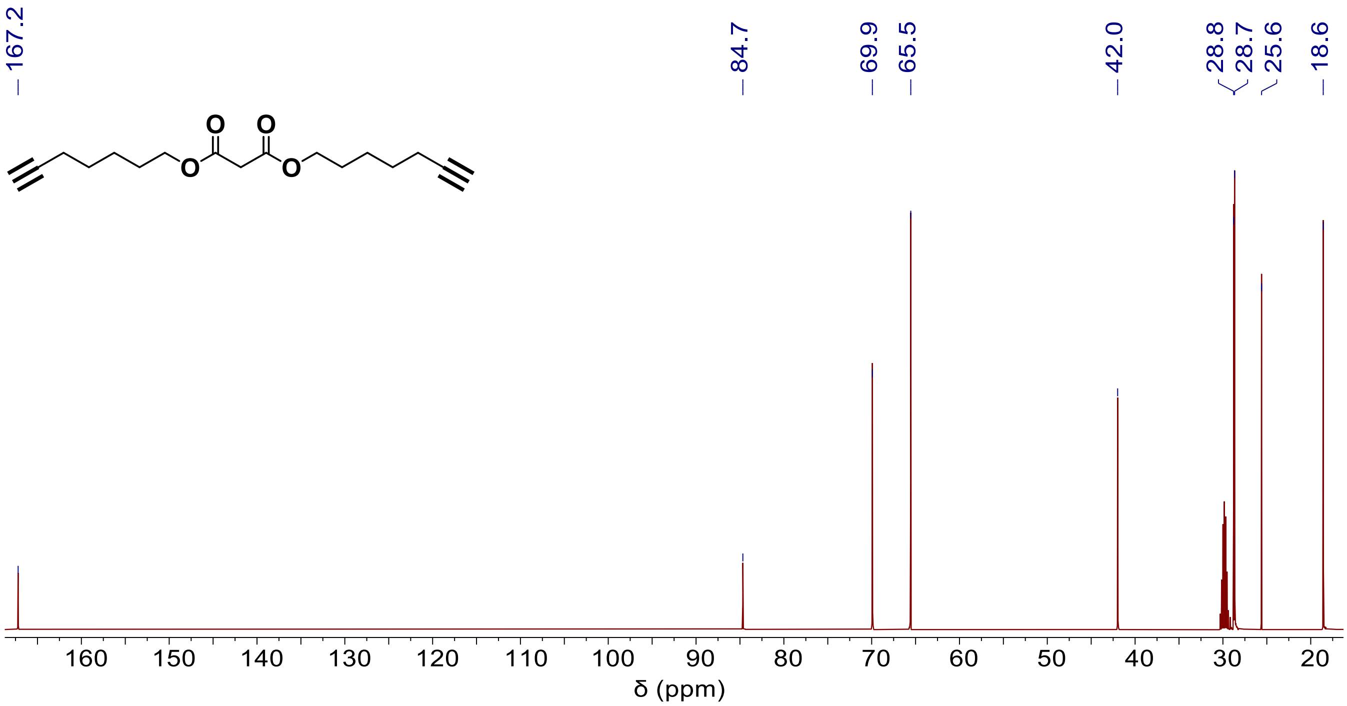

Figure S2. ^{13}C NMR spectrum of 2 in acetone-d_6 at 75 MHz.

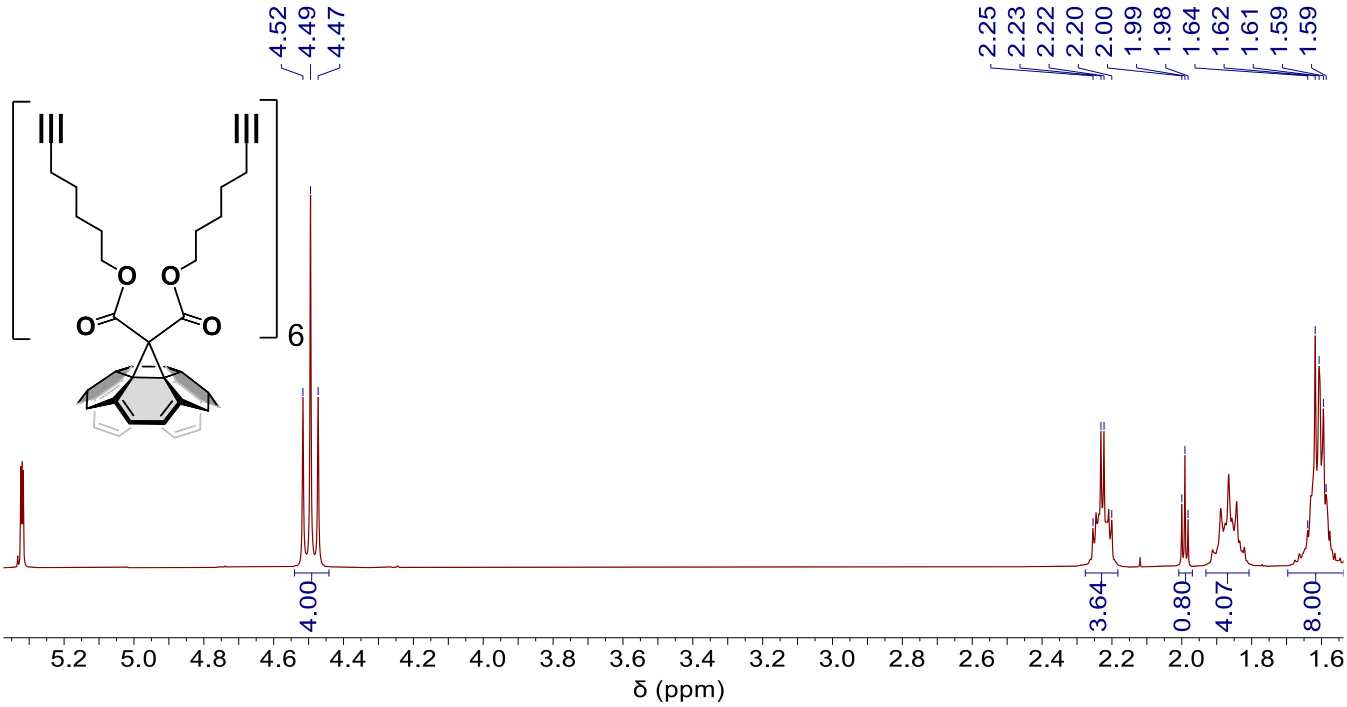

Figure S3. ^{1}H NMR spectrum of F-Alkyne$_{12}$ in CH_2Cl_2-d_2 at 500 MHz.

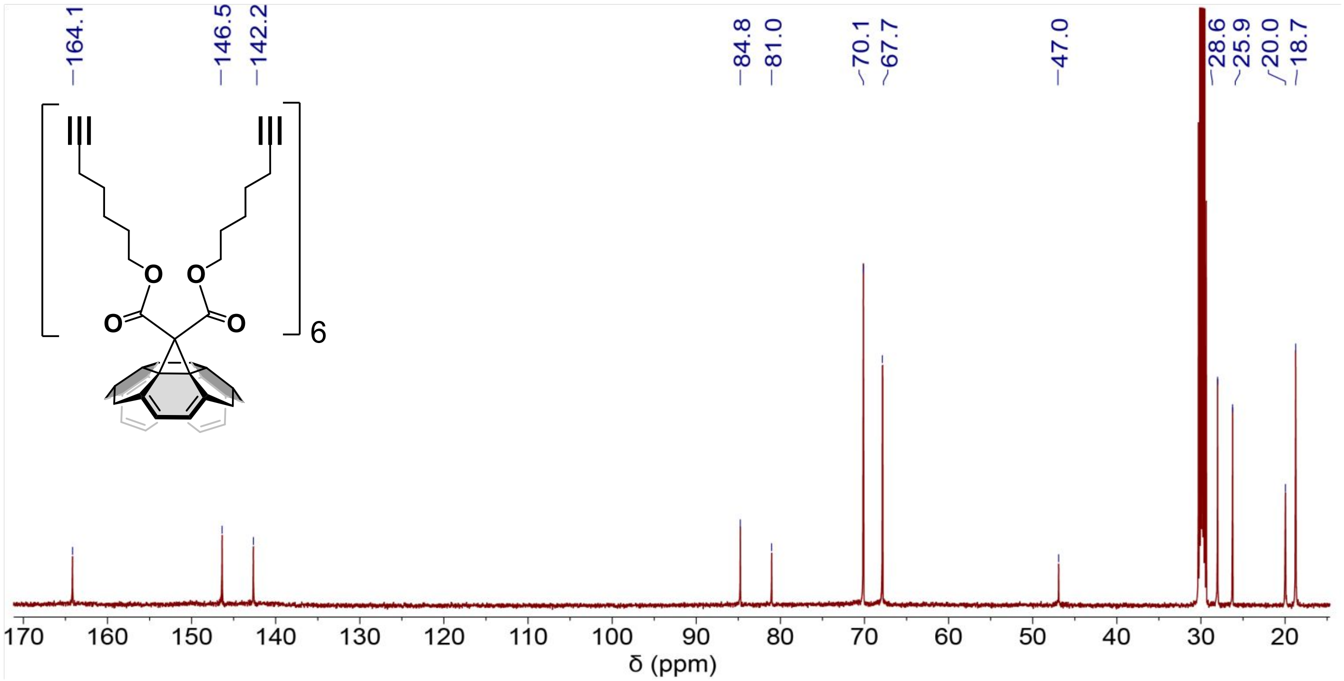

Figure S4. ^{13}C NMR spectrum of F-Alkyne$_{12}$ in acetone-d_6 at 126 MHz.

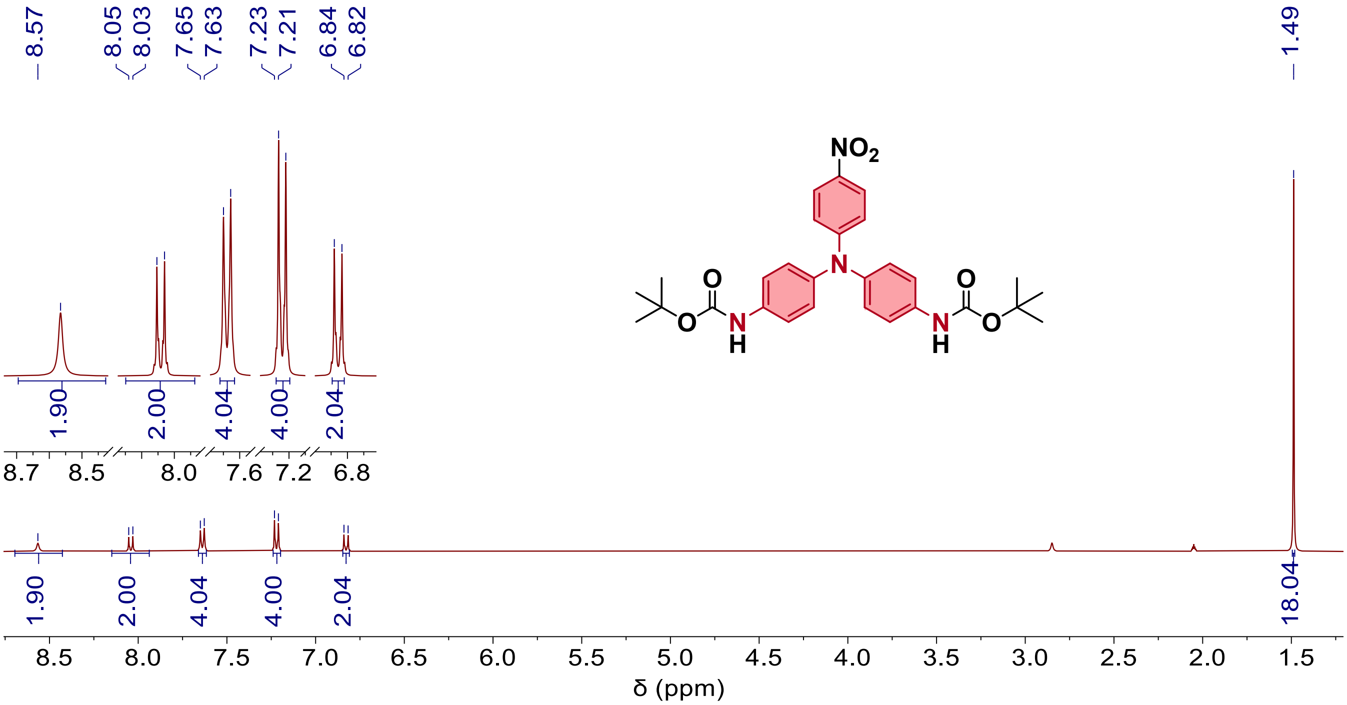

Figure S5. ^{1}H NMR spectrum of 4 in acetone-d_6 at 300 MHz.

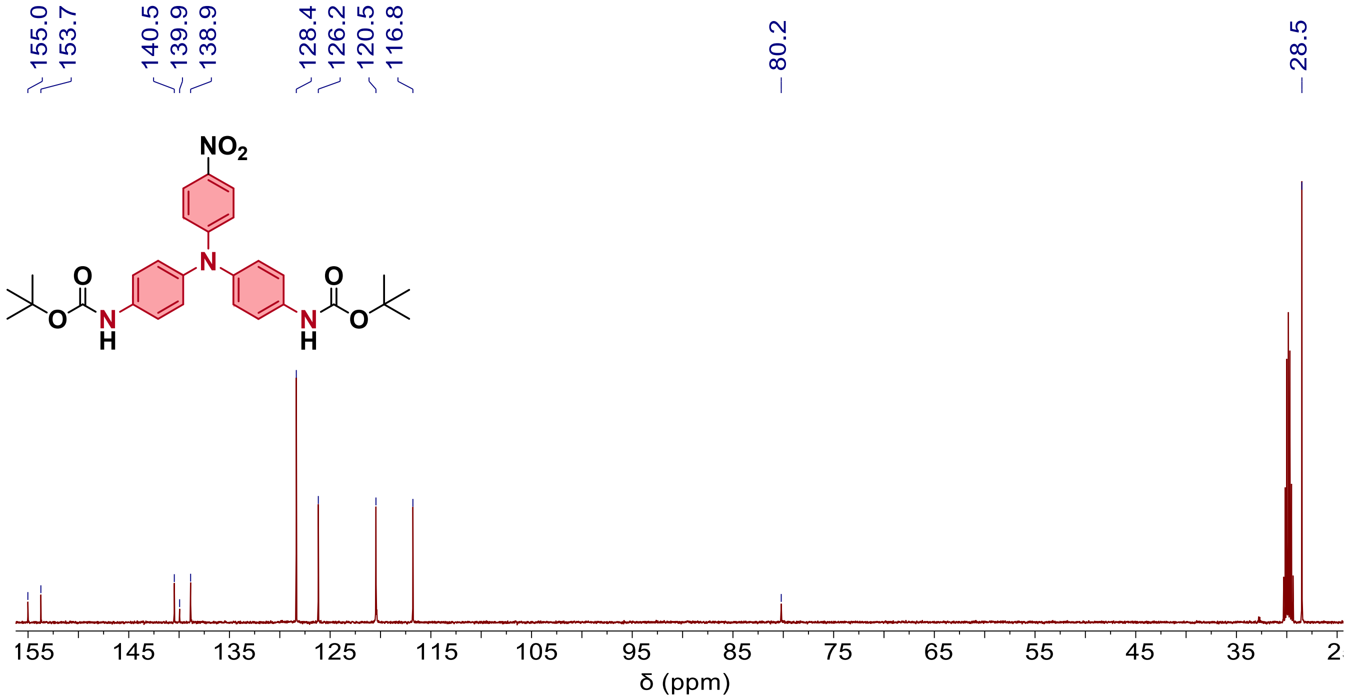

Figure S6. ^{13}C NMR spectrum of 4 in acetone-d_6 at 75 MHz.

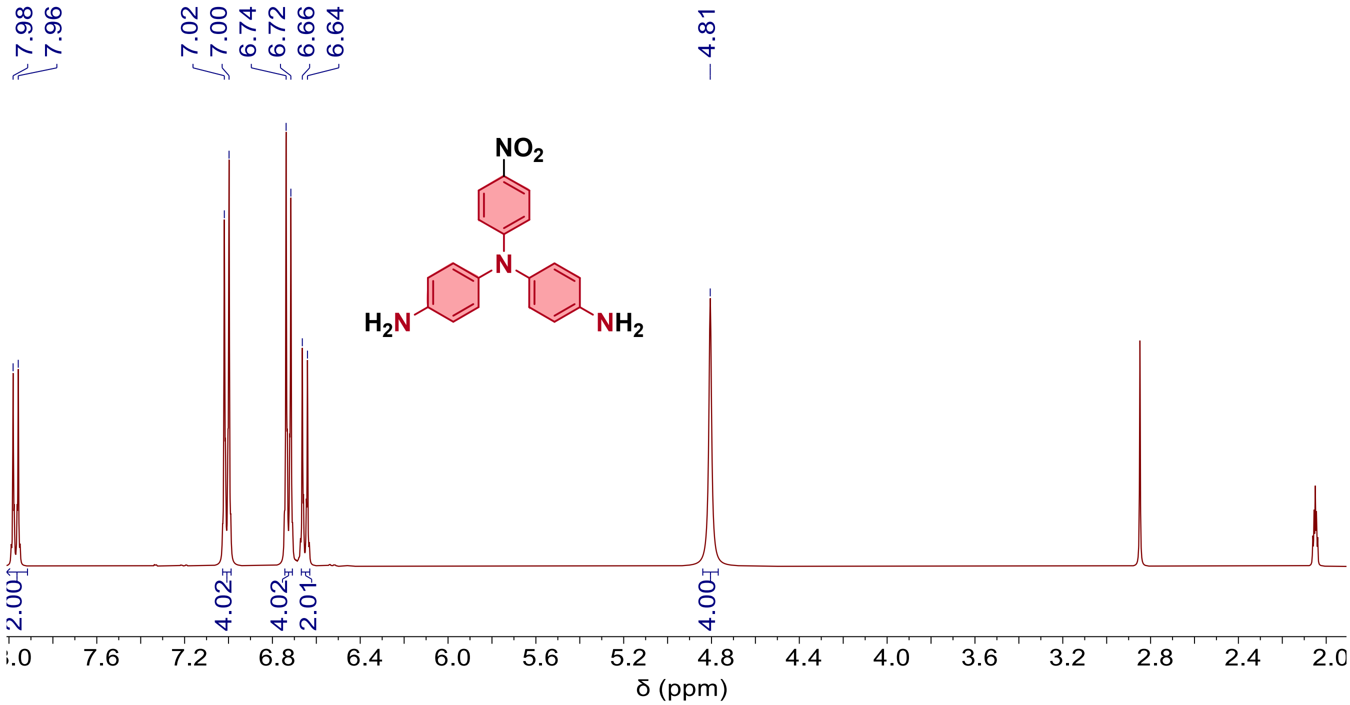

Figure S7. ^{1}H NMR spectrum of 5 in acetone-d_6 at 300 MHz.

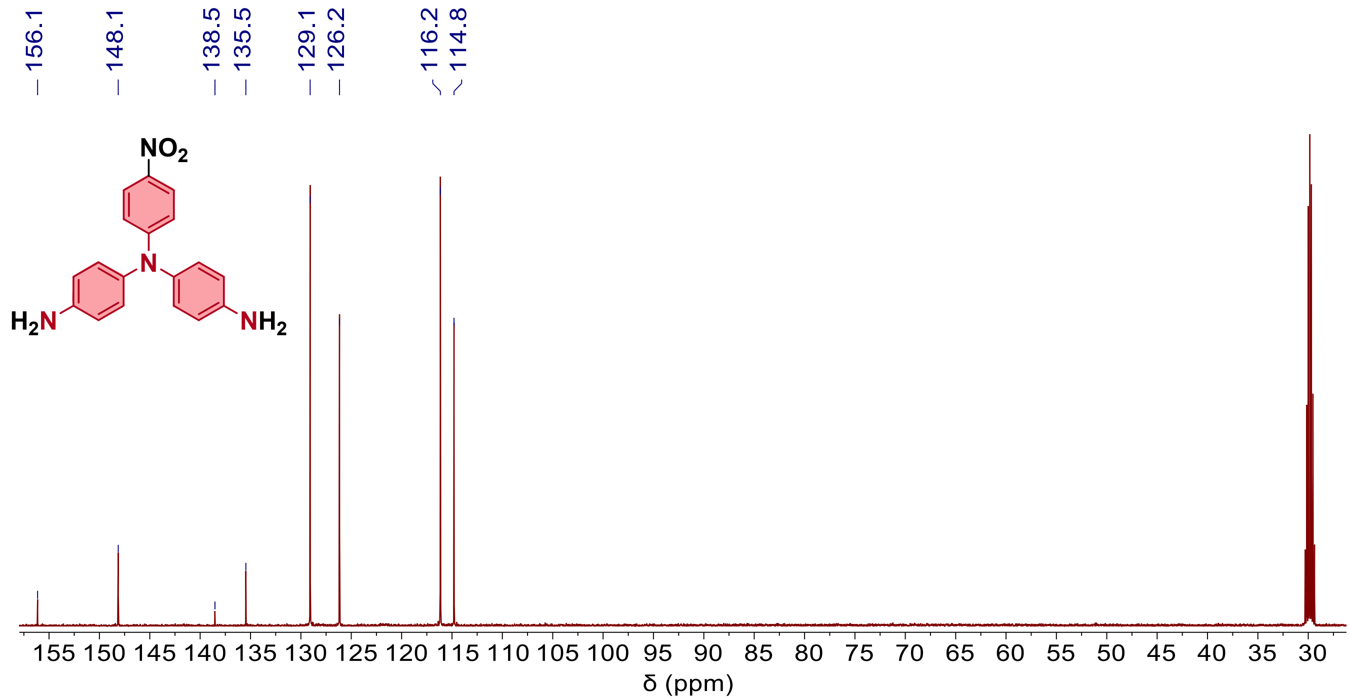

Figure S8. ^{13}C NMR spectrum of 5 in acetone-d_6 at 75 MHz.

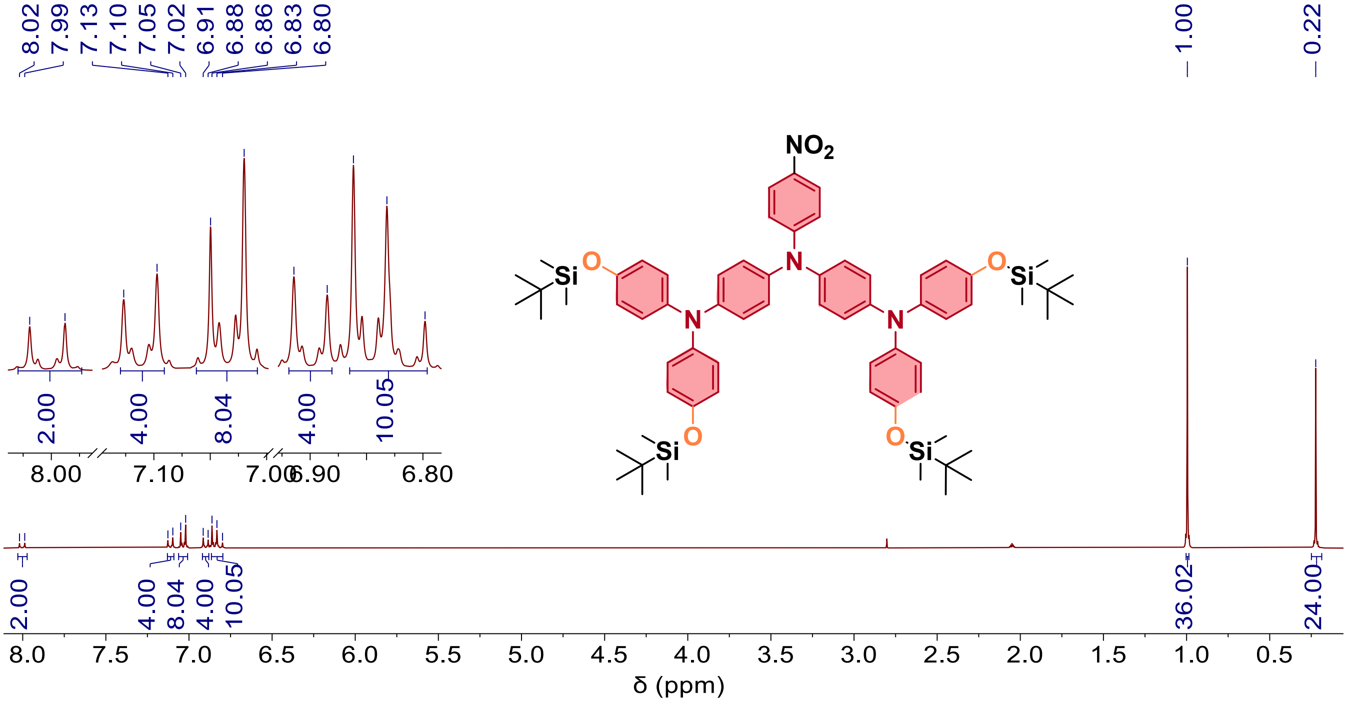

Figure S9. ^{1}H NMR spectrum of 6 in acetone-d_6 at 300 MHz.

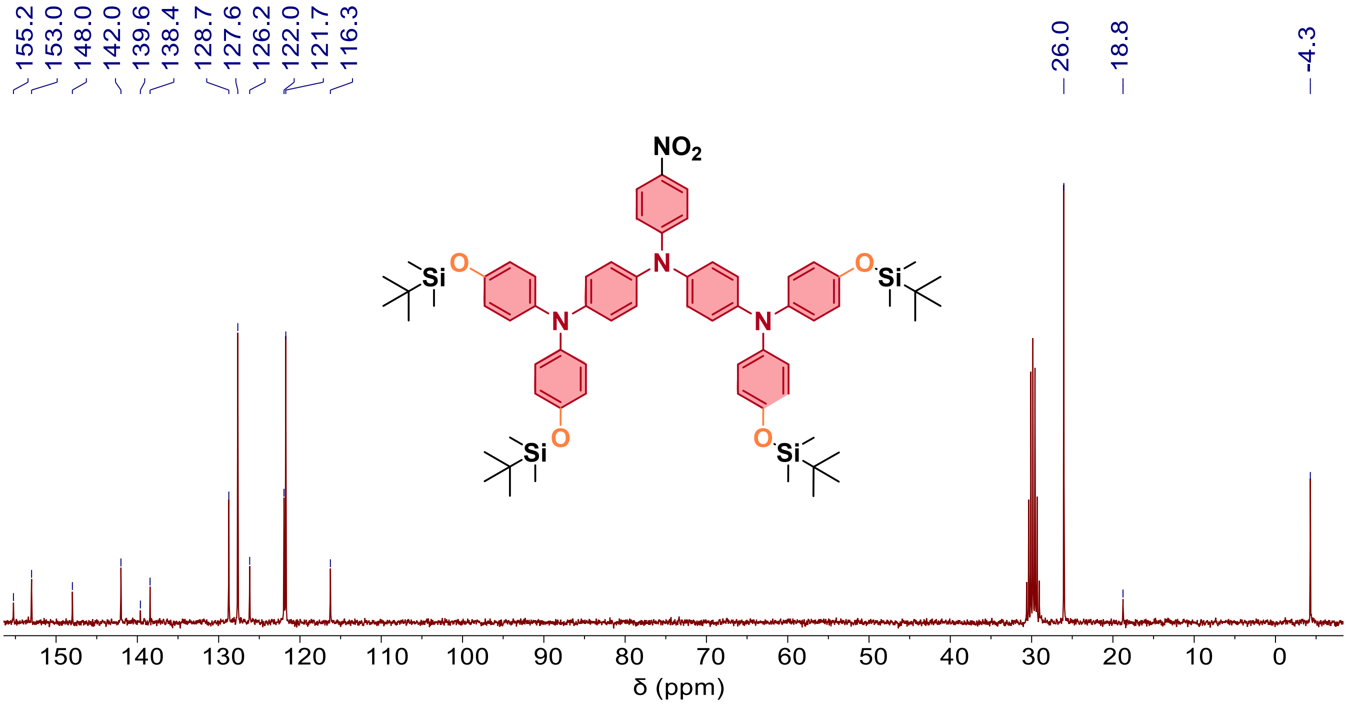

Figure S10. ^{13}C NMR spectrum of 6 in acetone-d_6 at 75 MHz.

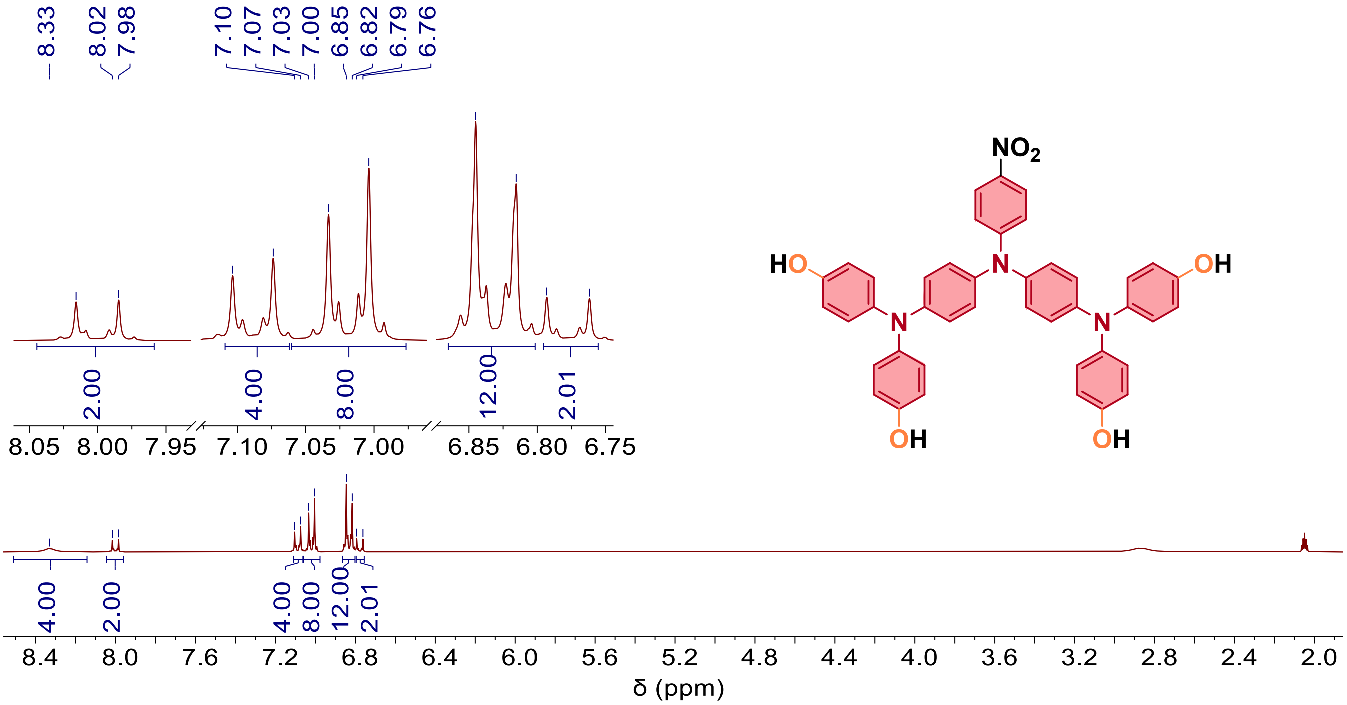

Figure S11. ¹H NMR spectrum of 7 in acetone-d_6 at 300 MHz.

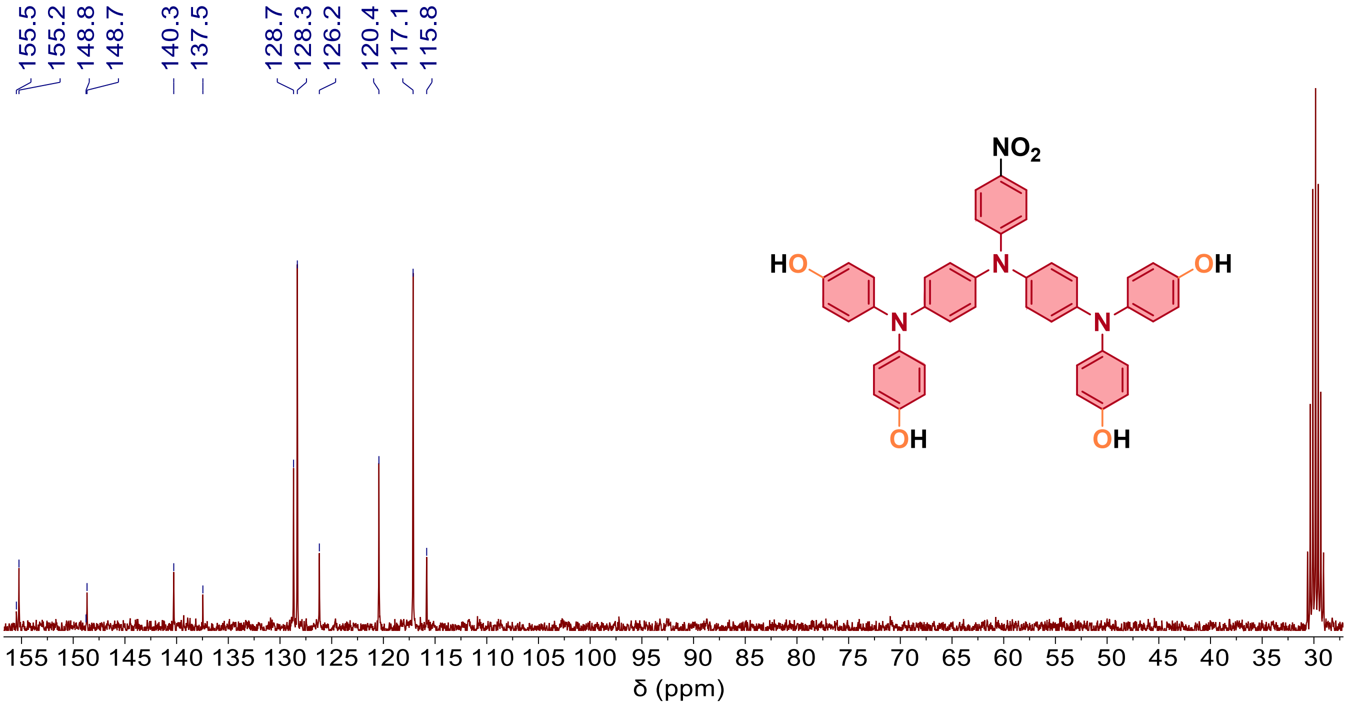

Figure S12. ¹³C NMR spectrum of 7 in acetone-d_6 at 75 MHz.

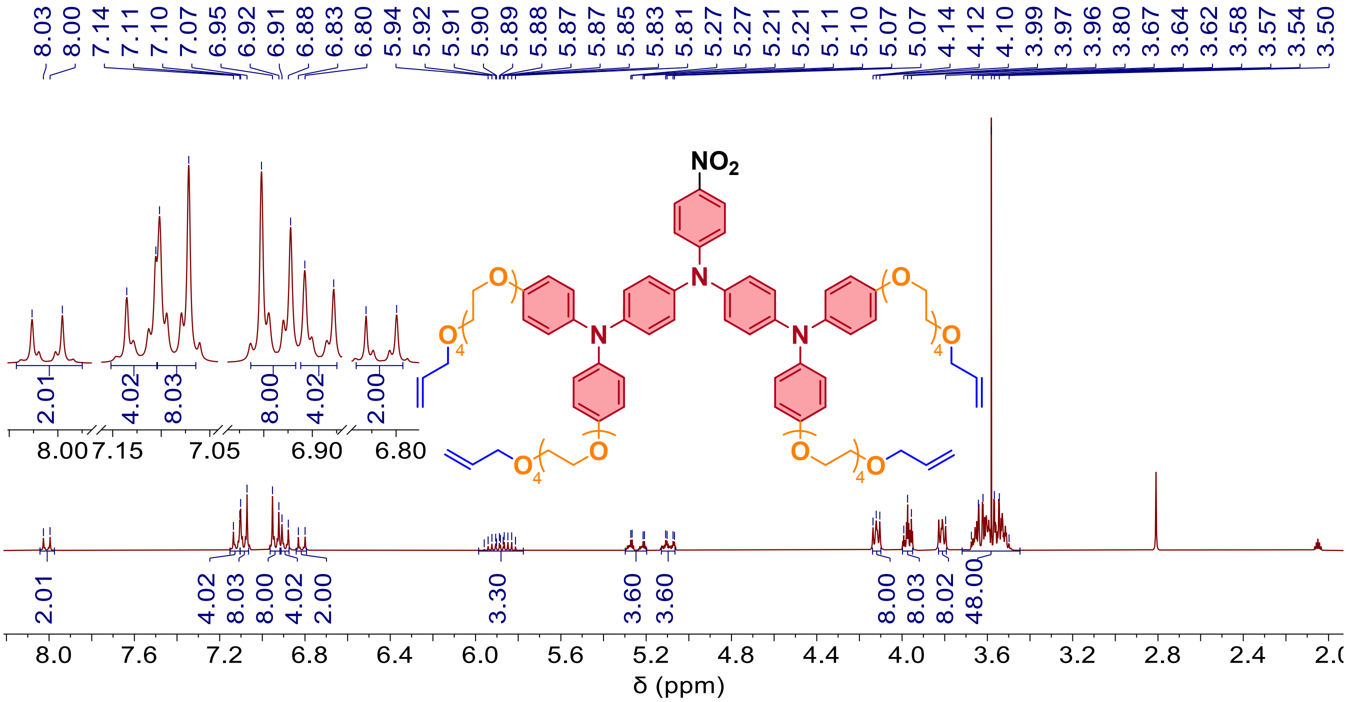

Figure S13. ^{1}H NMR spectrum of 8 in acetone-d_6 at 300 MHz.

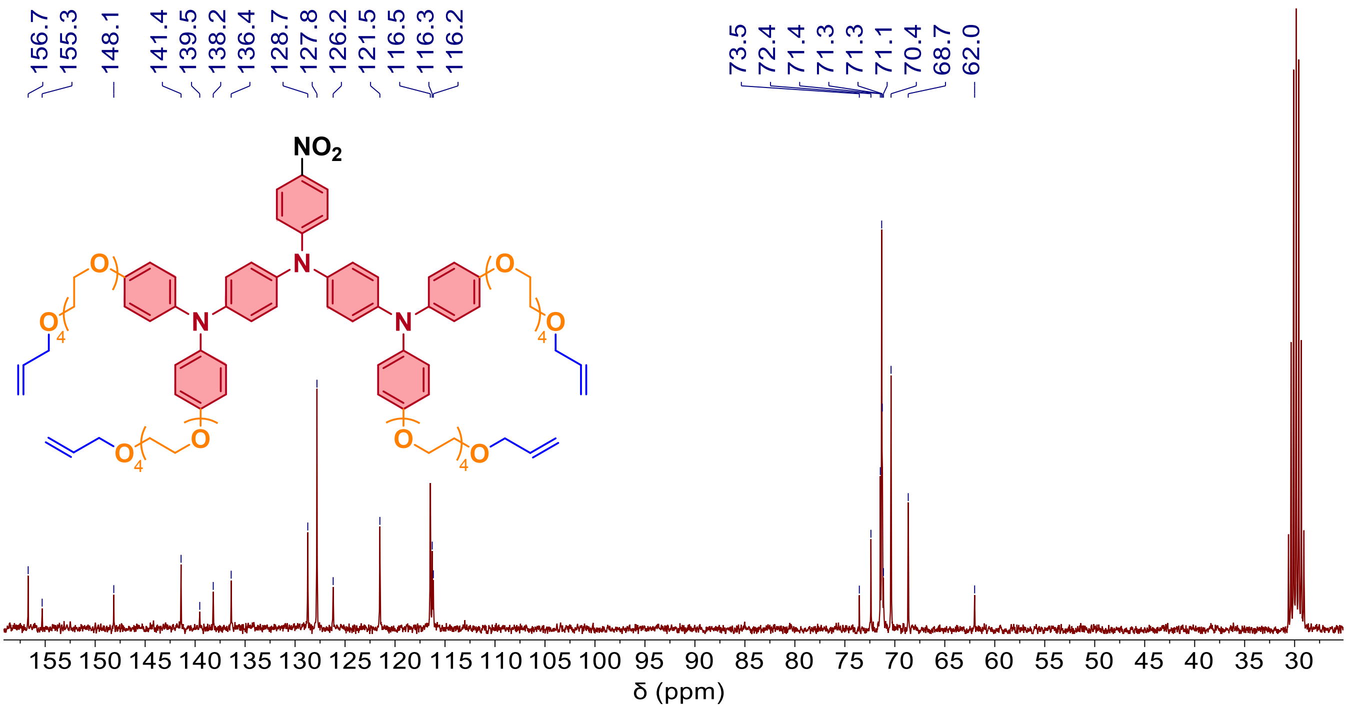

Figure S14. ^{13}C NMR spectrum of 8 in acetone-d_6 at 75 MHz.

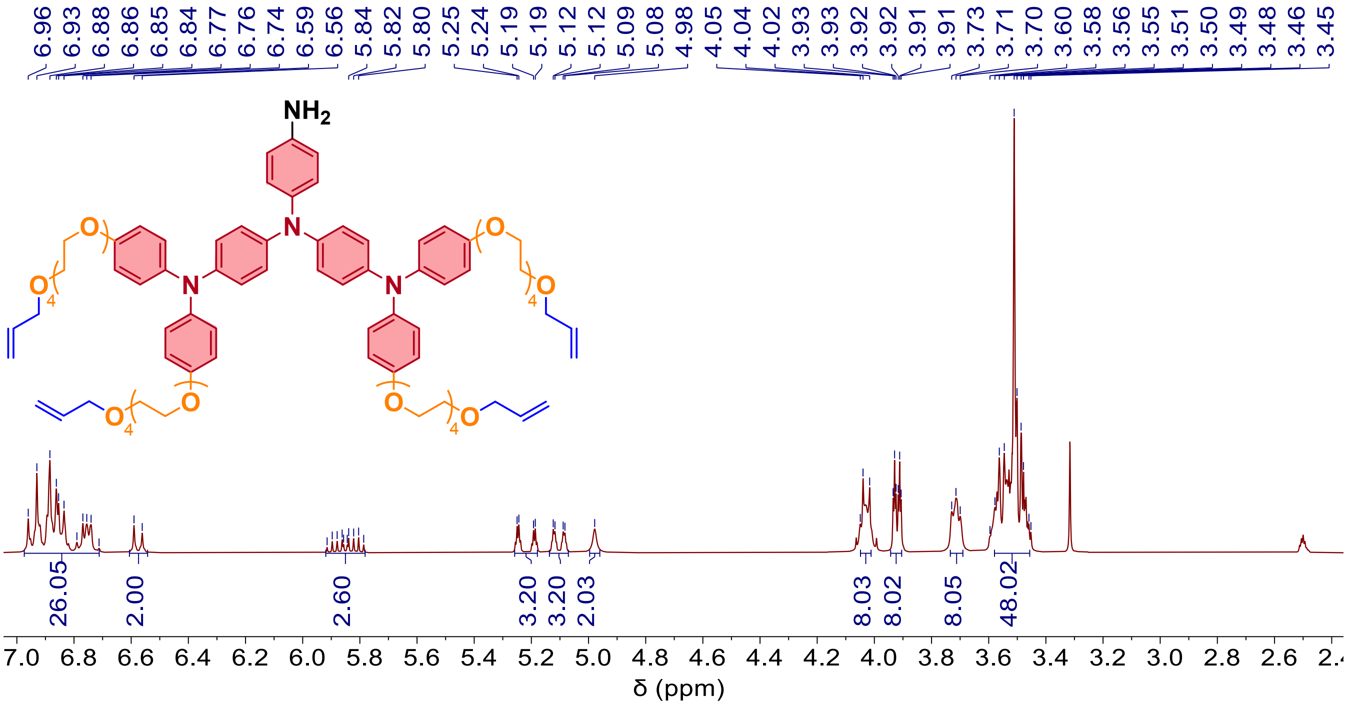

Figure S15. ^{1}H NMR spectrum of 9 in DMSO-d_6 at 300 MHz.

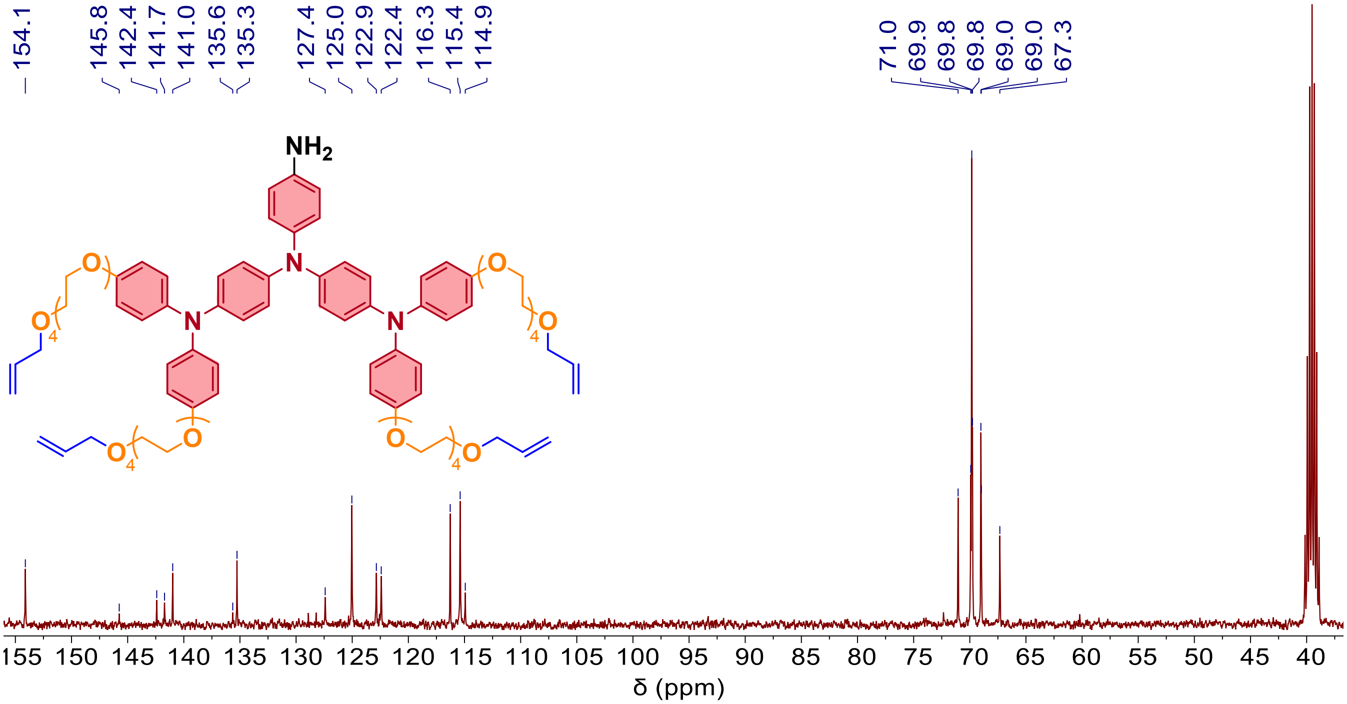

Figure S16. ^{13}C NMR spectrum of 9 in DMSO-d_6 at 75 MHz.

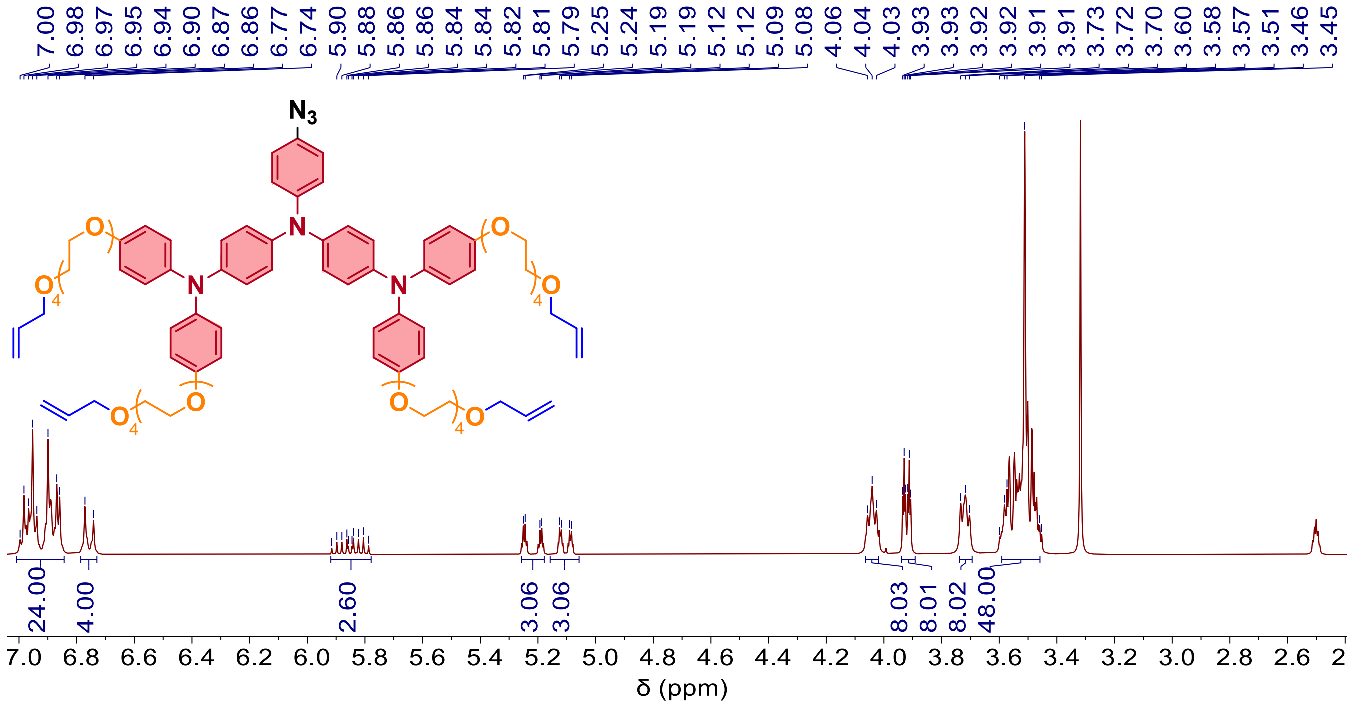

Figure S17. ^{1}H NMR spectrum of T-Azide in DMSO-d_6 at 300 MHz.

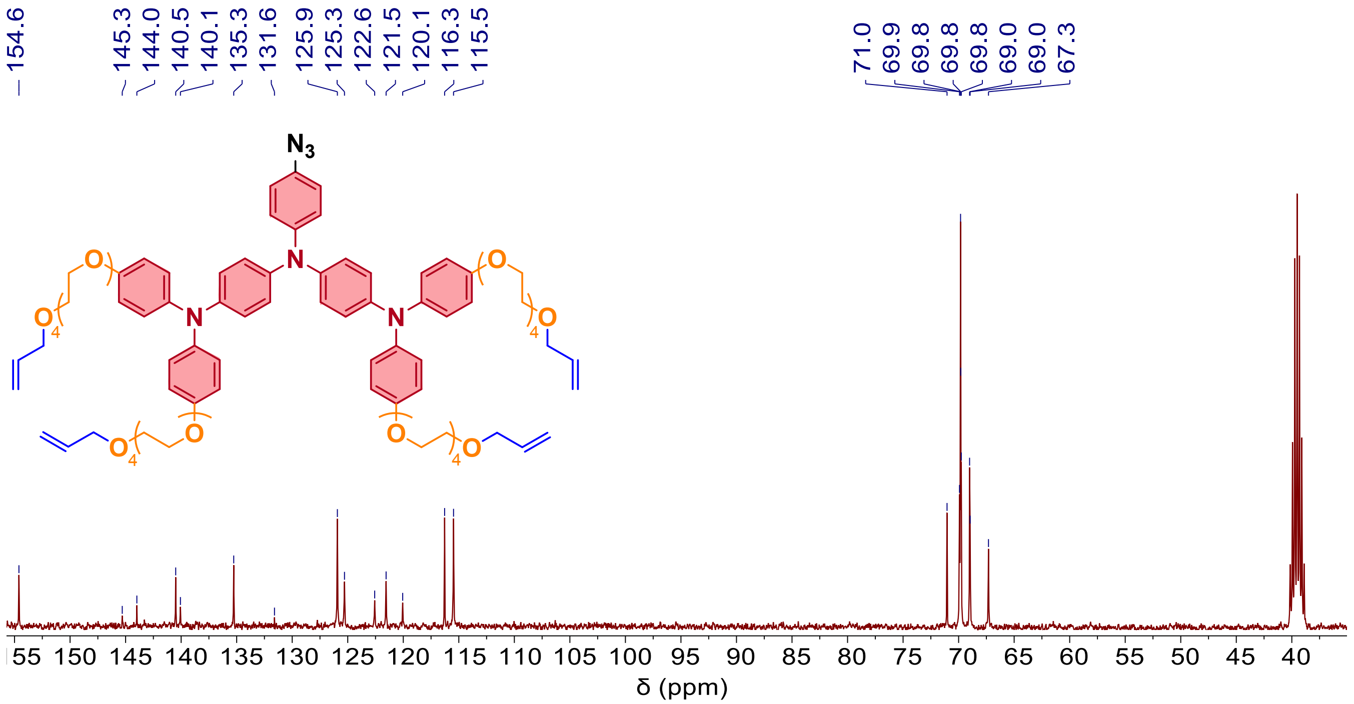

Figure S18. ^{13}C NMR spectrum of T-Azide in DMSO-d_6 at 75 MHz

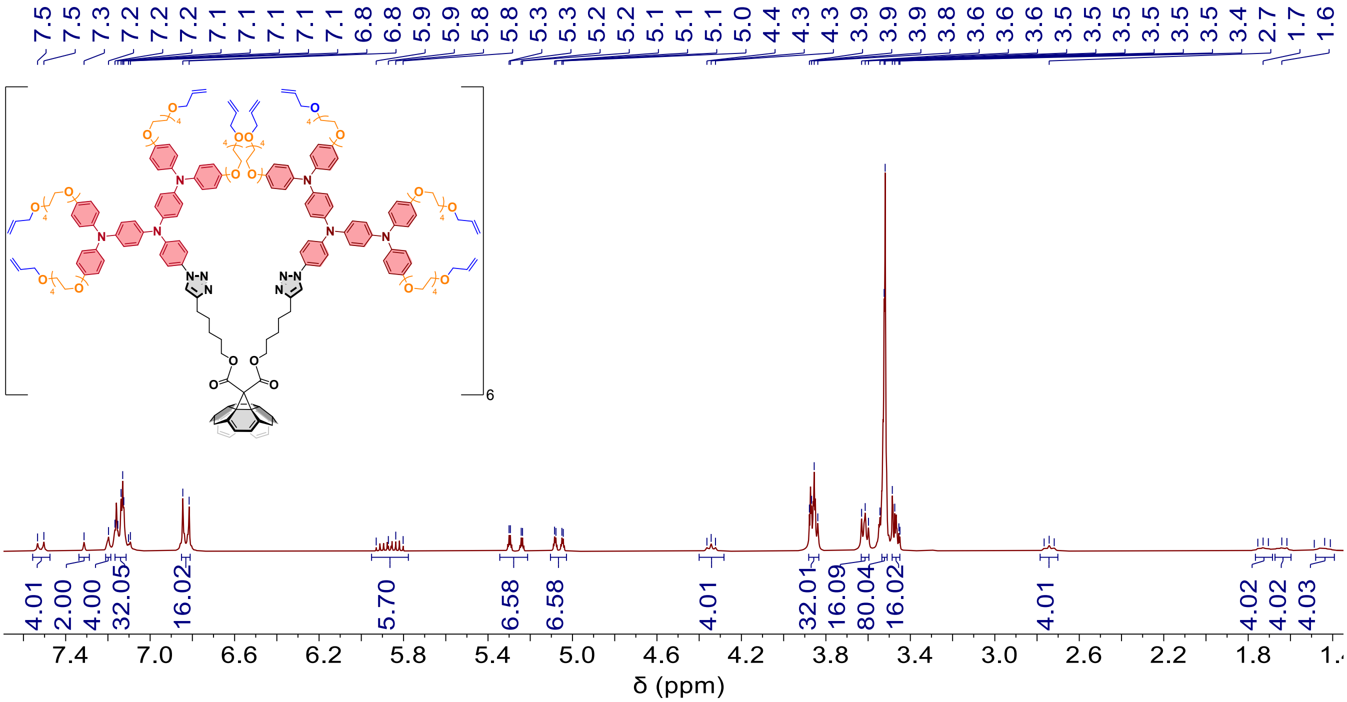

Figure S19. ^{1}H NMR spectrum of FT_{12} in benzene-d_6 at 500 MHz.

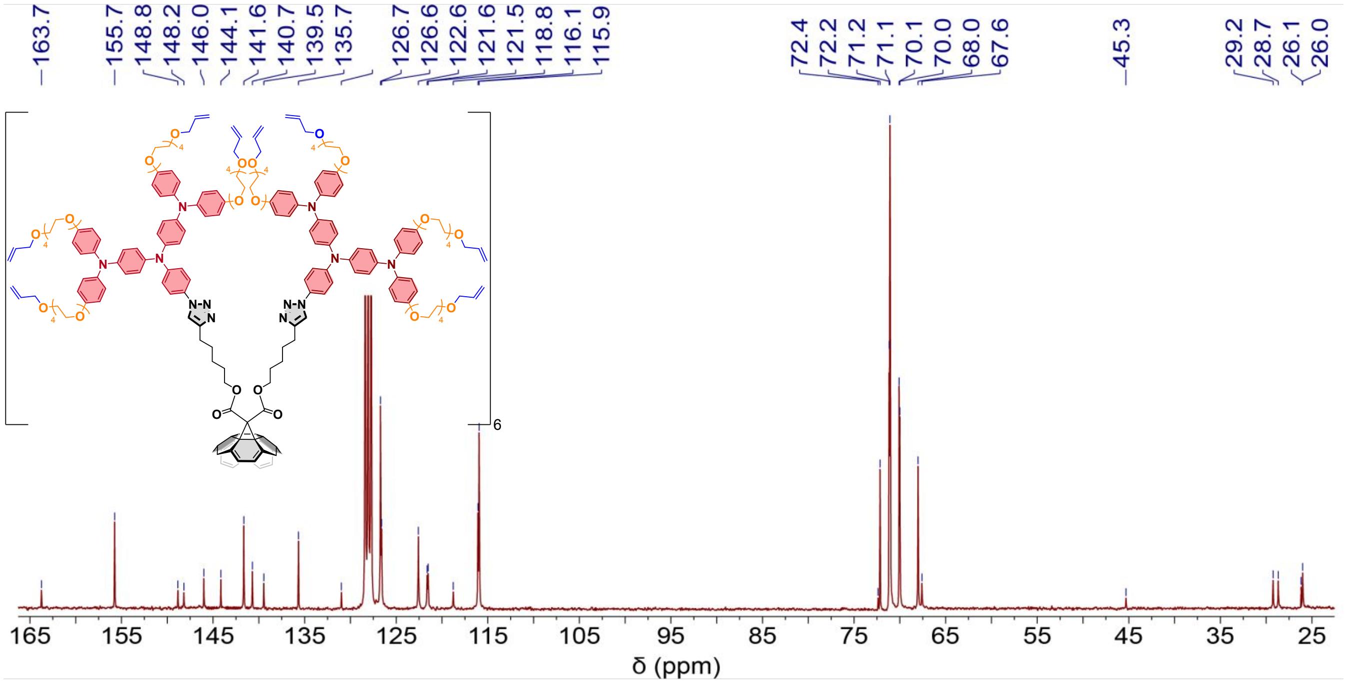

Figure S20. ^{13}C NMR spectrum of FT_{12} in benzene-d_6 at 126 MHz.

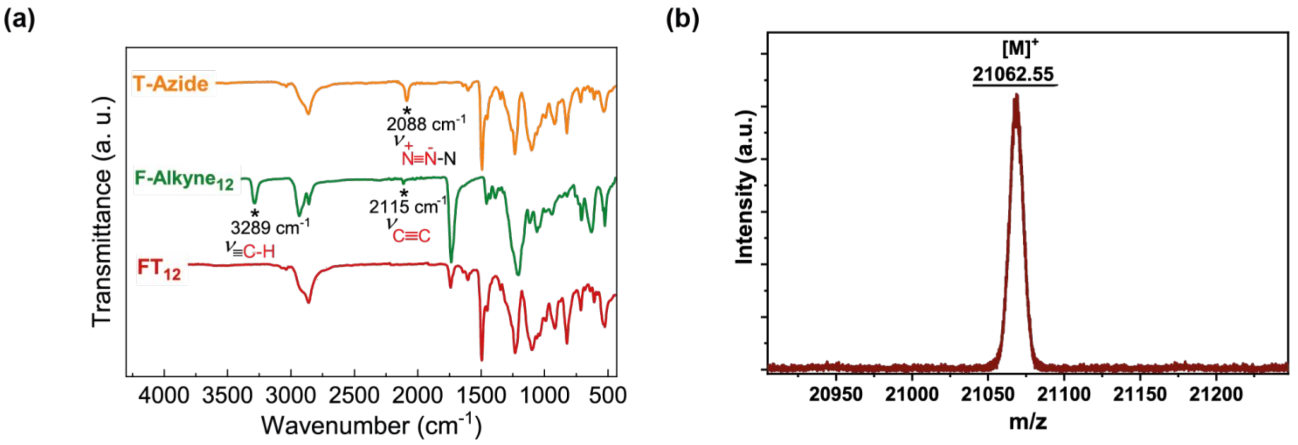

Figure S21. a) FTIR spectra of T-Azide, F-Alkyne and FT_{12}. b) MALDI-TOF mass spectrum of FT_{12}.

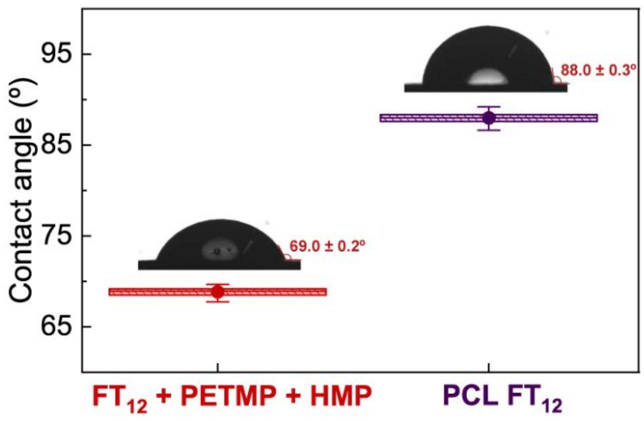

Figure S22. Statistical contact angle data.

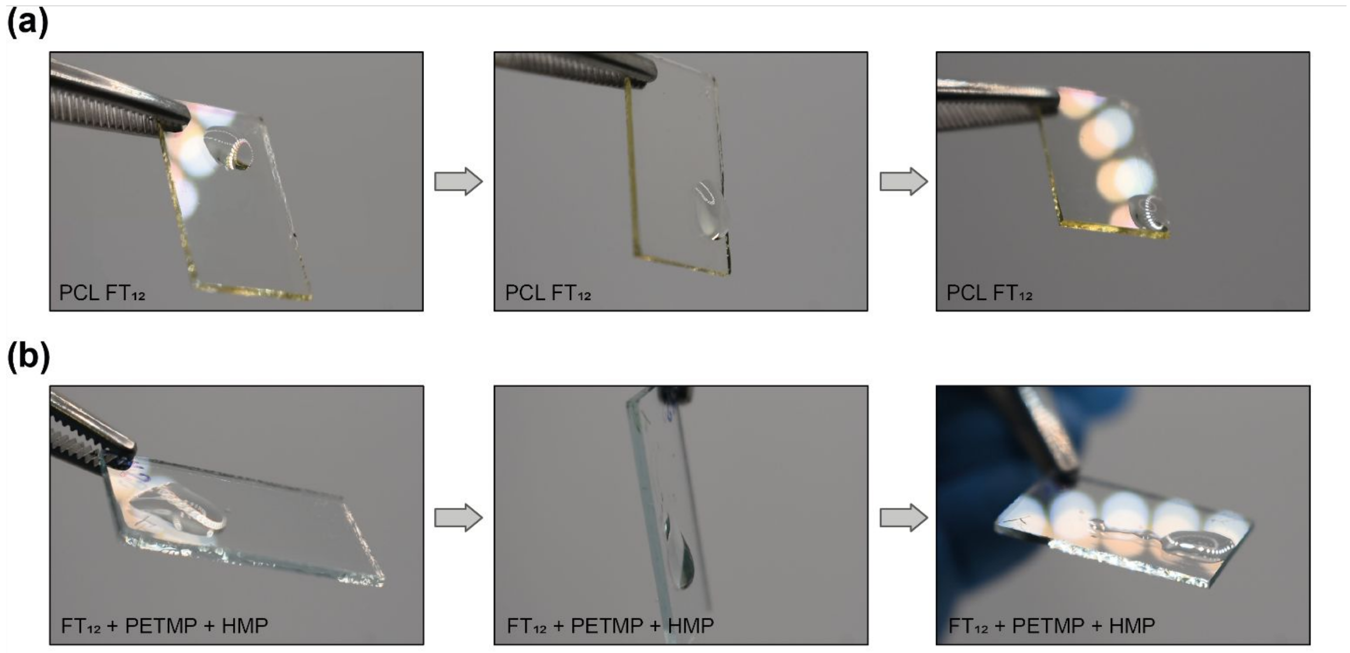

Figure S23. Video captures displaying the movement of a water droplet across a surface based on (a) PCL FT_{12} and (b) FT_{12} + PETMP + HMP.

2.2. Characterization of perovskite solar cells

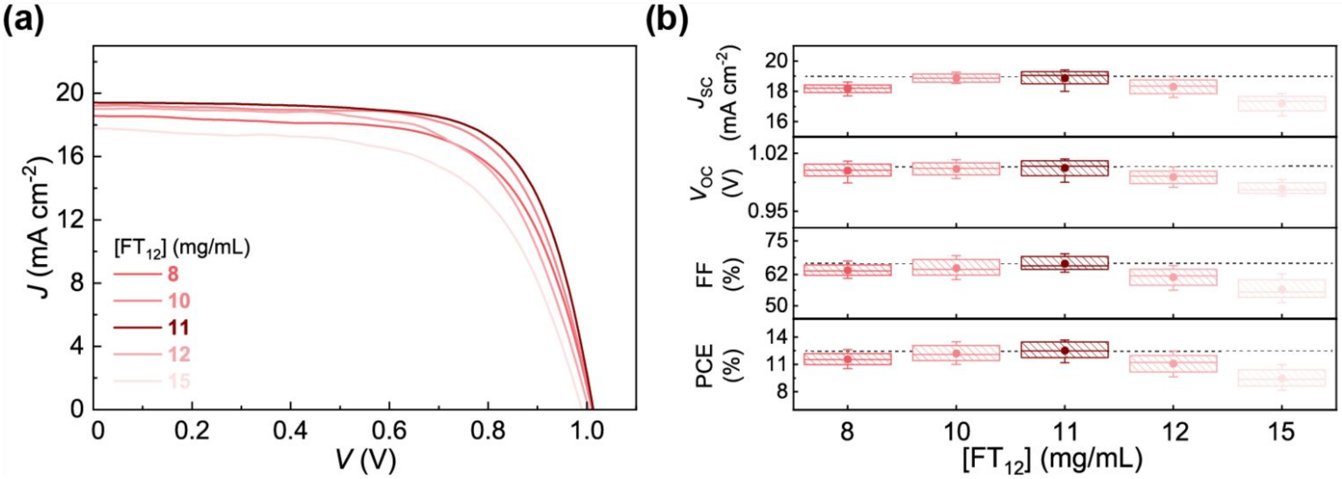

Figure S24. (a) *J-V* curves of the best-performing PSCs with HTLs prepared with different concentrations of FT_{12} precursor solution. (b) Statistical box plots of photovoltaic parameters from around 15 devices. The line of the graph has been positioned at the average value obtained using a concentration of 11 mg/mL.

Table S1. Best and average photovoltaic parameters of PSCs with HTLs prepared from different concentrations of FT_{12} precursor solution. (Statistical results obtained from around 15 devices per condition).

[FT_{12}] (mg/mL)	J_{SC} (mA cm^{-2})	V_{OC} (V)	FF (%)	PCE (%)
8	18.6<br>(18.2 ± 0.3)	1.01<br>(0.99 ± 0.01)	67<br>(64 ± 2)	12.6<br>(11.6 ± 0.8)
10	19.3<br>(18.9 ± 0.3)	1.01<br>(1.00 ± 0.01)	69<br>(65 ± 3)	13.5<br>(12.2 ± 0.9)
11	19.4<br>(18.9 ± 0.5)	1.01<br>(1.00 ± 0.01)	70<br>(66 ± 3)	13.7<br>(12.5 ± 0.9)
12	19.0<br>(18.3 ± 0.5)	1.00<br>(0.99 ± 0.01)	65<br>(61 ± 4)	12.5<br>(11.0 ± 0.9)
15	17.8<br>(17.2 ± 0.5)	0.99<br>(0.98 ± 0.01)	62<br>(56 ± 4)	11.0<br>(9.0 ± 0.8)

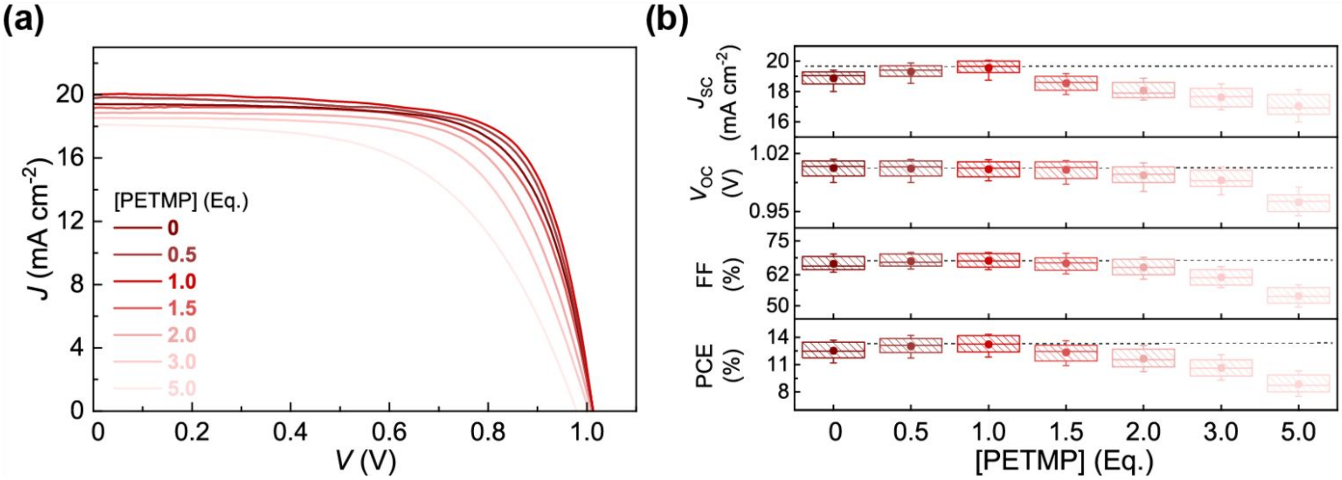

Figure S25. (a) *J-V* curves of the best-performing PSCs with HTLs consisting of FT_{12} and different molar equivalents of PETMP. (b) Statistical box plots of photovoltaic parameters from around 15 devices. The line of the graph has been positioned at the average value achieved with a PETMP concentration of 1 equivalent.

Table S2. Best and average photovoltaic parameters of PSCs with HTLs consisting of FT_{12} and different molar equivalents of PETMP. (Statistical results obtained from 15 devices per condition).

[PETMP] (Eq.)	J_{SC} (mA cm^{-2})	V_{OC} (V)	FF (%)	PCE (%)
0	19.4<br>(18.9 ± 0.5)	1.01<br>(1.00 ± 0.01)	70<br>(66 ± 3)	13.7<br>(12.5 ± 0.9)
0.5	19.9<br>(19.3 ± 0.5)	1.01<br>(1.00 ± 0.01)	71<br>(67 ± 2)	14.2<br>(13.0 ± 0.9)
1.0	20.1<br>(19.6 ± 0.5)	1.01<br>(1.00 ± 0.01)	71<br>(68 ± 3)	14.3<br>(13.2 ± 0.9)
1.5	19.2<br>(18.5 ± 0.5)	1.01<br>(1.00 ± 0.01)	70<br>(66 ± 3)	13.6<br>(12.0 ± 1.0)
2.0	18.9<br>(18.1 ± 0.6)	1.01<br>(0.99 ±0.01)	69<br>(65 ± 3)	13.1<br>(12.0 ± 1.0)
3.0	18.5<br>(17.6 ± 0.6)	1.00<br>(0.99 ± 0.01)	65<br>(61 ± 3)	12.1<br>(11.0 ± 1.0)
5.0	18.1<br>(17.0 ± 0.7)	0.98<br>(0.96 ± 0.01)	58<br>(54 ± 3)	10.3<br>(9.0 ± 1.0)

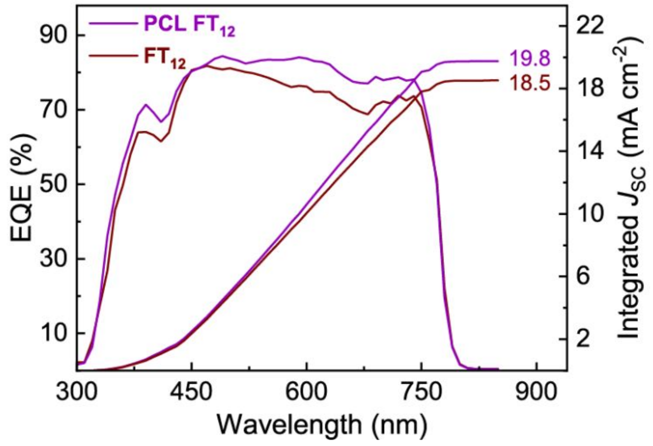

Figure S26. EQE spectra and integrated J_{SC} of FT12 and PCL FT_{12}-based PSCs.

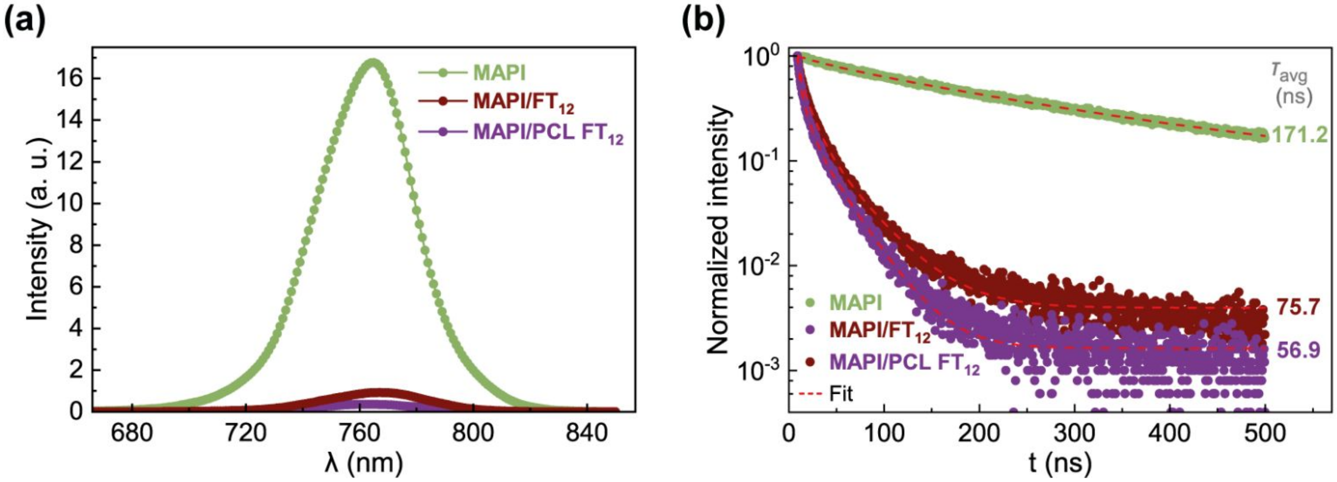

Figure S27. (a) Steady-state PL spectra and (b) TRPL lifetime curves of MAPI/HTL substrates.

The time-resolved photoluminescence (TRPL) decays were fitted using the following bi-exponential equation:[9]

$$\tau(t) = A_1 e^{-\left(\frac{t}{\tau 1}\right)} + A_2 e^{-\left(\frac{t}{\tau 2}\right)}$$

Where A_1 and A_2 are the amplitude of the radiative decay lifetime and τ represents the lifetime values. The average lifetime (τ_{avg}) was calculated as follows the equation: $\tau_{avg} = (A_1\tau_1^2 + A_2\tau_2^2)/(A_1\tau 1 + A_2\tau 2)$. The fitting parameters are collected in Table S3.

Table S3. The fitting parameters of photoluminescence decay curves for MAPI/HTL substrates.

Substrate	A_1 (%)	A_2 (%)	τ_1 (ns)	τ_2 (ns)	τ_{avg} (ns)
MAPI	4.6	95.4	33.1 ± 1.8	172.5 ± 1.9	171.2
MAPI/FT_{12}	44.1	55.9	31.1 ± 0.5	88.1 ± 1.4	75.7
MAPI/PCL FT_{12}	30	70	20.7 ± 0.4	62.1 ± 0.5	56.9

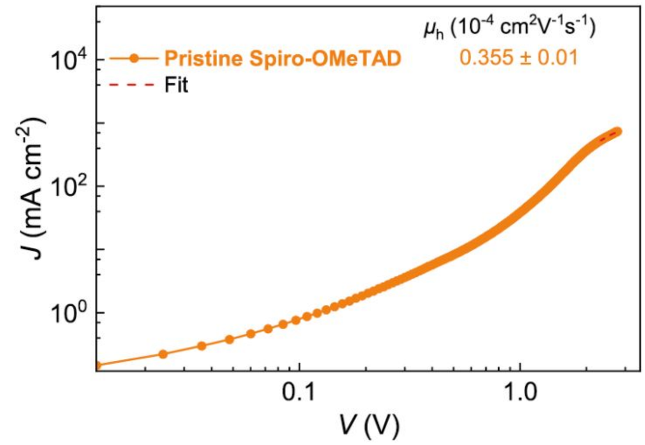

Figure S28. Hole mobility (μ_h) of the pristine spiro-OMeTAD layer.

REFERENCES

(1) Delavaux-Nicot, B.; Ben Aziza, H.; Nierengarten, I.; Minh Nguyet Trinh, T.; Meichsner, E.; Chessé, M.; Holler, M.; Abidi, R.; Maisonhaute, E.; Nierengarten, J.-F. A Rotaxane Scaffold for the Construction of Multiporphyrinic Light-Harvesting Devices. *Chemistry – A European Journal* 2018, *24* (1), 133–140. https://doi.org/10.1002/chem.201704124.

(2) Collavini, S.; Völker, S. F.; Cabrera-Espinoza, A.; Martínez, M. A.; De Cózar, A.; San Felices, L.; Sánchez, L.; Delgado, J. L. Triarylamine Enriched Organostannoxane Drums: Synthesis, Optoelectrochemical Properties, Association Studies, and Gelation Behavior. *Inorg. Chem.* 2022, *61* (9), 4046–4055. https://doi.org/10.1021/acs.inorgchem.1c03761.

(3) Li, H.; Haque, Sk. A.; Kitaygorodskiy, A.; Meziani, M. J.; Torres-Castillo, M.; Sun, Y.-P. Alternatively Modified Bingel Reaction for Efficient Syntheses of C_{60} Hexakis- Adducts. *Org. Lett.* 2006, *8* (24), 5641–5643. https://doi.org/10.1021/ol062391d.

(4) Samples, E. M.; Schuck, J. M.; Joshi, P. B.; Willets, K. A.; Dobereiner, G. E. Synthesis and Properties of *N*-Arylpyrrole-Functionalized Poly(1-Hexene-Alt-CO). *Macromolecules* 2018, *51* (22), 9323–9332. https://doi.org/10.1021/acs.macromol.8b01629.

(5) Ogawa, T.; Ohta, K.; Iijima, T.; Suzuki, T.; Ohta, S.; Endo, Y. Synthesis and Biological Evaluation of *p*-Carborane Bisphenols and Their Derivatives: Structure–Activity Relationship for Estrogenic Activity. *Bioorg. Med. Chem.* 2009, *17* (3), 1109–1117. https://doi.org/10.1016/j.bmc.2008.12.044.

(6) Dickschat, A. T.; Behrends, F.; Bühner, M.; Ren, J.; Weiß, M.; Eckert, H.; Studer, A. Preparation of Bifunctional Mesoporous Silica Nanoparticles by Orthogonal Click Reactions and Their Application in Cooperative Catalysis. *Chemistry – A European Journal* 2012, *18* (52), 16689–16697. https://doi.org/10.1002/chem.201200499.

(7) Kitamura, M.; Yano, M.; Tashiro, N.; Miyagawa, S.; Sando, M.; Okauchi, T. Direct Synthesis of Organic Azides from Primary Amines with 2-Azido-1,3-dimethylimidazolinium Hexafluorophosphate. *Eur. J. Org. Chem.* 2011, *2011* (3), 458–462. https://doi.org/10.1002/ejoc.201001509.

(8) Uceta, H.; Cabrera-Espinoza, A.; Barrejón, M.; Sánchez, J. G.; Gutierrez-Fernandez, E.; Kosta, I.; Martín, J.; Collavini, S.; Martínez-Ferrero, E.; Langa, F.; Delgado, J. L. *P*-Type Functionalized Carbon Nanohorns and Nanotubes in Perovskite Solar Cells. *ACS Appl. Mater. Interfaces* 2023. https://doi.org/10.1021/acsami.3c07476.

(9) Zhao, Z. Q.; You, S.; Huang, J.; Yuan, L.; Xiao, Z. Y.; Cao, Y.; Cheng, N.; Hu, L.; Liu, J. F.; Yu, B. H. Molecular Modulator for Stable Inverted Planar Perovskite Solar Cells with Efficiency Enhanced by Interface Engineering. *J. Mater. Chem. C* 2019, *7* (31), 9735–9742. https://doi.org/10.1039/C9TC03259B.
